# The many flavours of halogen bonds – message from experimental electron density and Raman spectroscopy

**DOI:** 10.1107/S205322961901132X

**Published:** 2019-08-22

**Authors:** Ruimin Wang, Janine George, Shannon Kimberly Potts, Marius Kremer, Richard Dronskowski, Ulli Englert

**Affiliations:** a Institute of Inorganic Chemistry, RWTH Aachen University, Landoltweg 1, Aachen 52056, Germany; b Institute of Molecular Science, Shanxi University, Taiyuan, Shanxi 030006, People’s Republic of China; c Institute of Condensed Matter and Nanosciences, Chemin des Étoiles 8/L7.03.01, Louvain-la-Neuve 1348, Belgium; d Jlich-Aachen Research Alliance (JARA-HPC), RWTH Aachen University, Aachen 52056, Germany; e Hoffmann Institute of Advanced Materials, Shenzhen Polytechnic, 7098 Liuxian Blvd, Shenzhen, People’s Republic of China

**Keywords:** halogen bonds, experimental electron density, AIM analysis, crystal engineering, crystal structure, Raman spectroscopy

## Abstract

Experimental electron-density studies based on high-resolution diffraction experiments allow halogen bonds between heavy halogens to be classified. The topological properties of the electron density in Cl⋯Cl contacts vary smoothly as a function of the inter­action distance. The situation is less straightforward for halogen bonds between iodine and small electronegative nucleophiles, such as nitro­gen or oxygen, where the electron density in the bond critical point does not simply increase for shorter distances. The number of successful charge–density studies involving iodine is small, but at least individual examples for three cases have been observed. (*a*) Very short halogen bonds between electron-rich nucleophiles and heavy halogen atoms resemble three-centre–four-electron bonds, with a rather symmetric heavy halogen and without an appreciable σ hole. (*b*) For a narrow inter­mediate range of halogen bonds, the asymmetric electronic situation for the heavy halogen with a pronounced σ hole leads to rather low electron density in the (3,−1) critical point of the halogen bond; the properties of this bond critical point cannot fully describe the nature of the associated inter­action. (*c*) For longer and presumably weaker contacts, the electron density in the halogen bond critical point is only to a minor extent reduced by the presence of the σ hole and hence may be higher than in the aforementioned case. In addition to the electron density and its derived properties, the halogen–carbon bond distance opposite to the σ hole and the Raman frequency for the associated vibration emerge as alternative criteria to gauge the halogen-bond strength. We find exceptionally long C—I distances for tetra­fluoro­diiodo­benzene molecules in cocrystals with short halogen bonds and a significant red shift for their Raman vibrations.

## Abstract 2

The culprit is the σ-hole: short I⋯N halogen bonds do not necessarily come with high electron density in their (3,−1) critical point. The I—C geometry and Raman spectroscopy complement information from electron density.

## Introduction to halogen bonds   

**Figure 1:**
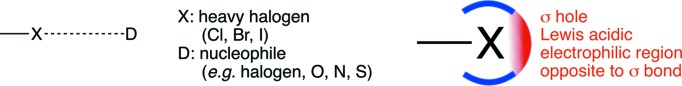
Halogen bonds and the σ-hole.

The term ‘halogen bond’ denotes a short contact between a Lewis base *D* and a heavy halogen *X* (I, Br or Cl) acting as electrophile (Hassel, 1970 ▸; Metrangolo & Resnati, 2001 ▸); a schematic overview is provided in Fig. 1 ▸. More generally, halogen bonds may be understood as a special case of contacts in which a nucleophile approaches the electrophilic region of a neighbouring atom, so-called σ-hole inter­actions (Brinck *et al.*, 1992 ▸, 1993 ▸; Politzer *et al.*, 2017 ▸; George *et al.*, 2014 ▸).

The nucleophilic atom *D* usually corresponds to N, O or Cl, but other elements carrying a lone pair that is sufficiently exposed to the periphery and accessible to short contacts may also qualify as electron-density donors, *e.g.* sulfur (Şerb *et al.*, 2015 ▸). In the most popular case in which the halogen *X* is engaged in only one bond, its σ hole forms opposite to it, implying a very pronounced directionality. The short contacts *X*⋯*D*, which we nowadays address as halogen bonds, have not gone unnoticed by chemical crystallographers. We only mention two early examples here in which the authors explicitly point out short inter­molecular distances. In the very first volume of *Acta Crystallographica*, E. Archer commented on the short inter­molecular I⋯O distances of 2.72 Å between neighbouring IO_2_ groups in 1-chloro-4-iodyl­benzene (Archer, 1948 ▸). The 1969 Nobel prize winner Hassel and co-workers (Borgen *et al.*, 1962 ▸) reported I⋯N contacts of 2.93 Å between neighbouring mol­ecules of 3-iodo­propiolo­nitrile, *i.e.* cyano- and iodo-substituted ethyne. In parallel with the idea of ‘crystal engineering’ (Desiraju, 1995 ▸), the number of pub­lished papers devoted to halogen bonds has markedly increased, from about 150 per year in the 1970s and 1980s to about 1000 per year in the last decade. A full account of the historic developments of the halogen bond and its applications in supra­molecular chemistry is beyond the scope of this feature article and has been provided in a recent review (Cavallo *et al.*, 2016 ▸).

An analysis of the Cambridge Structural Database (CSD; Groom *et al.*, 2016 ▸) proves the existence of short and strongly directional contacts about heavy halogen atoms; intuition suggests two approaches to verify the inter­action model described above. (*a*) Computational methods show the anisotropic charge distribution about *X* and have often been used to justify experimental crystal structures, both for classical mol­ecular crystals and biological structures (Wolters *et al.*, 2014 ▸; Ford & Ho, 2016 ▸). (*b*) In principle, X-ray diffraction is not limited to atomic resolution but may map the experimental electron density in more detail (Coppens, 1997 ▸). We here use high-resolution X-ray diffraction data to analyse and classify halogen bonds.

## Charge density of halogen bonds   

Not only *X*, the inter­action partner with the σ hole, but also the Lewis base *D* (the halogen-bond acceptor) in Fig. 1 ▸ may be a halogen atom. Experimental charge–density studies on such short inter­halogen contacts will be addressed in §2.1, whereas *X*⋯*D* contacts between a heavy halogen and an O or N atom will be discussed in §2.2.

### Results on inter­halogen contacts   

**Figure 2:**
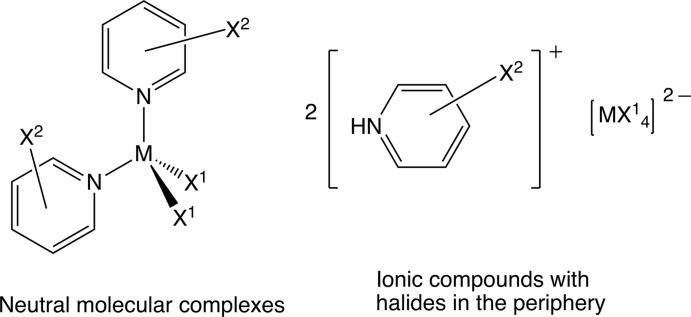
Two classes of compounds in which short inter­halogen contacts are likely to occur.

**Figure 3:**
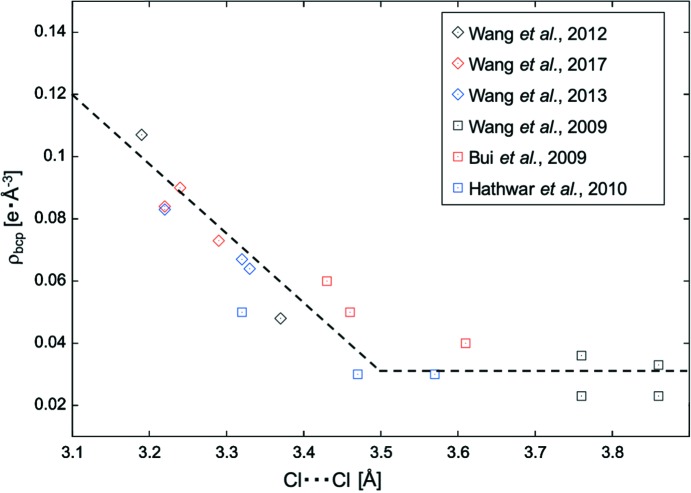
Graphical summary of electron density ρ in the Cl⋯Cl bond critical point (bcp) *versus* inter­molecular distance in short inter­chlorine contacts; dashed lines have been drawn to guide the eye and do not imply any fit.

Already in 1963, Sakurai *et al.* (1963 ▸) noted that *R*—*X*⋯*X*—*R* contacts (*X* = halogen atom) occur preferentially according to two distinct geometries. A geometric explanation for this directionality has become known as polar flattening (Nyburg & Faerman, 1985 ▸); experimental charge–density studies are more recent. In order to better understand the protopypic structure of the diatomic heavy halides, Zhou and co-workers (Tsirelson *et al.*, 1995 ▸) combined experimental data for crystalline Cl_2_ and the topology of the Laplacian for an isolated dichlorine mol­ecule; Richard Bader was a co-author of this article. A few years later, Boese and colleagues (Seppelt *et al.*, 2004 ▸) communicated the crystal structure of chlorine fluoride, ClF, which was unexpectedly dominated by short inter­chlorine contacts rather than by dipole inter­actions. This study of a short inter­halogen contact represents an early example in which the analysis of the electron density was completely based on experimental data. In contrast to this early charge–density report, which dealt with Cl⋯Cl inter­actions shorter than 3.1 Å, experimental electron-density studies by the groups of Espinosa (Bui *et al.*, 2009 ▸) and Guru Row (Hathwar *et al.*, 2010 ▸) covered longer inter­molecular Cl⋯Cl contacts. In order to induce shorter inter­chlorine distances in preferably stable crystalline solids and enlarge the experimental evidence on halogen bonds, we followed two approaches (Fig. 2 ▸).

(*a*) Complexes of divalent metal cations with halide ligands *X* ^1^ and halide(*X* ^2^)-substituted pyridines feature halogen atoms in their periphery and form mol­ecular crystals in which short inter­halogen contacts are very likely. (*b*) Halogen(*X* ^2^)-substituted pyridinium cations and tetra­halo(*X* ^1^)metallate anions aggregate to salts, subtending hydrogen and halogen bonds. In both target classes of compounds, short inter­halogen contacts occur with high frequency. Several among the latter ionic compounds formed crystals of only standard quality (Wang & Englert, 2017 ▸) but others proved sufficient for an experimental electron-density study (Wang *et al.*, 2017 ▸). Together with earlier results from our group (Wang *et al.*, 2009 ▸, 2012 ▸, 2013 ▸) and those mentioned above (Bui *et al.*, 2009 ▸; Hathwar *et al.*, 2010 ▸), we can compile 18 examples of inter­chlorine contacts for which details of the experimental charge densities and properties of the bond critical points (bcps) are available. In Fig. 3 ▸, the electron density in the Cl⋯Cl bcps has been plotted as a function of the inter­chlorine distance.

We can identify two regions in Fig. 3 ▸. (*a*) The electron density in the bcp increases with decreasing Cl⋯Cl distances shorter than *ca* 3.5 Å, the van der Waals distance (Bondi, 1964 ▸). This trend is not surprising because the short contacts are subtended by atoms of the same element type, *i.e.* similar electronegativities and atomic radii, and the bcp can be expected roughly at the mid-point between the participating atoms. (*b*) For inter­chlorine distances longer than 3.5 Å, the electron density in the bcp remains low and does not significantly vary as a function of the contact distance. In this region, the observed values for ρ are most likely to be too small to allow conclusions concerning the strength and nature of the underlying inter­molecular contacts (Kamiński *et al.*, 2014 ▸).

### Results for I⋯N and I⋯O halogen bonds   

A very different situation is encountered for halogen bonds between the heavy halides and small electronegative Lewis basic atoms. In the context of experimental charge–density studies, Coppens (1977 ▸) coined the term ‘suitability’ for the ratio between valence and total electrons. With respect to this qualifier, short contacts to chlorine are the most attractive targets and the presence of iodine represents a particular challenge: crystals of very good quality and X-ray data of high redundancy will be required for a successful charge–density study. In terms of inter­action strength, however, the sequence I > Br > Cl is accepted (Cavallo *et al.*, 2016 ▸) and halogen bonds involving iodine can be associated with clearer polarization features and a more pronounced σ hole. We note that the halogen atom in-between these extremes, bromine, does not represent an attractive compromise, at least if the diffraction experiments are conducted with Mo *K*α radiation: absorption represents a major challenge for accurate diffraction experiments, and the linear absorption coefficient for the element bromine is significantly higher than for its heavier congener. 1,2,4,5-Tetra­fluoro-3,6-di­iodo­benzene (TFDIB) represents a particularly well-suited halogen-bond donor; it has been widely employed in crystal engineering. Bianchi and co-workers have performed high-resolution diffraction experiments to assess the experimental electron density in TFDIB cocrystals with short I⋯N [2.7804 (8) Å; Bianchi *et al.*, 2003 ▸] and I⋯O [2.7253 (10) Å; Bianchi *et al.*, 2004 ▸] contacts. We have already mentioned that they find a slightly lower electron density in the shorter halogen bond. In the context of our systematic work on ditopic ligands (Kremer & Englert, 2018 ▸), we have been able to investigate the charge density of a cocrystal between a substituted tris­(acetyl­acetonato)alumin­ium(III) complex and TFDIB (Merkens *et al.*, 2013 ▸); it involved I⋯O distances of 3.026 (6) and 3.157 (2) Å, and an I⋯N contact of 2.833 (3) Å.

In addition to these TFDIB adducts, we addressed a hypervalent iod­oxy compound, the so-called Togni reagent I (Kieltsch *et al.*, 2007 ▸). It is employed for the electrophilic transfer of a tri­fluoro­methyl group and features inter­molecular O⋯I contacts of 2.9822 (9) Å in the solid state. Our high-resolution diffraction experiment (Wang *et al.*, 2018*b* ▸) confirmed predictions concerning the σ hole (Kirshenboim & Kozuch, 2016 ▸) and the approach of a nucleophile as an important step in the suggested mechanism (Sala *et al.*, 2014 ▸, 2015 ▸). 70 years after Archer’s observation (Archer, 1948 ▸) of short I⋯O contacts between neighbouring iod­oxy groups, our *bona fide* first experimental charge density for a hypervalent iodine derivative provided crystallographic evidence for the charge distribution about the halogen bond behind these inter­actions.

**Figure 4:**
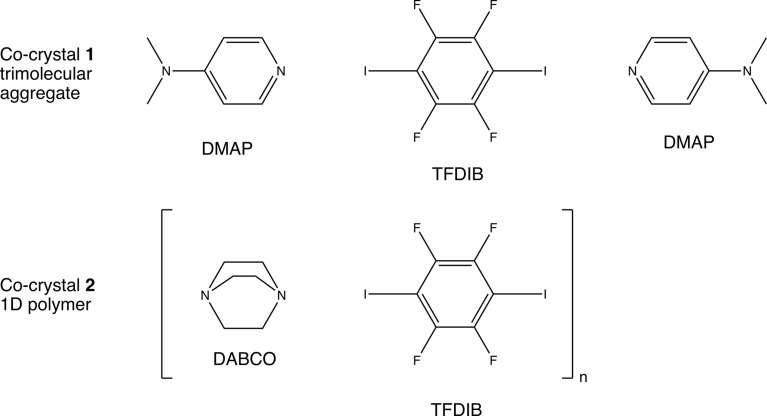
Target compounds with very short N⋯I contacts.

The above-mentioned charge–density studies revealed electron densities for the bcps in the I⋯*D* (*D* = N and O) halogen bonds with 0.24 > ρ_bcp_ > 0.08 e Å^−3^. We wanted to extend the contact range between TFDIB iodine and a suitable halogen-bond acceptor *D*, preferably Pearson-softer (Pearson, 1963 ▸) nitro­gen, to significantly shorter distances and investigate the electron density associated with these halogen bonds. In order to reliably obtain well-ordered crystalline solids suitable for high-resolution X-ray diffraction, we screened the CSD and identified compounds **1** and **2** shown in Fig. 4 ▸ as the most promising candidates.

The cocrystal of TFDIB with 4-(di­methyl­amino)­pyridine (DMAP), **1**, was first structurally characterized by Karadakov, Bruce and co-workers (Roper *et al.*, 2010 ▸). The composition of the solid is TFDIB(DMAP)_2_, with a trimolecular aggregate on a crystallographic centre of inversion. The I atom is engaged in a very short contact of 2.6622 (4) Å to the N atom of DMAP, a particularly nucleophilic pyridine derivative. The original authors did not only investigate short halogen bonds but also addressed the mechanochemical synthesis for this and related systems; we will come back to this aspect in §2.5. The 1:1 cocrystal formed by TFDIB and di­aza­bicyclo­octane (DABCO), **2**, features chains of alternating constituents, with two symmetry-independent N⋯I halogen bonds [2.7386 (11) and 2.7457 (10) Å]. Its structure has been reported three times based on intensity data with standard resolution (Bolte, 2004 ▸; Cinčić *et al.*, 2008 ▸; Syssa-Magalé *et al.*, 2014 ▸). The results of our charge–density studies on the very short I⋯N halogen bonds in **1** (Wang *et al.*, 2018*a* ▸) and **2** (this work) do not fit into a more general picture analogous to that encountered for inter­chlorine contacts (Fig. 3 ▸). Rather, the halogen bond in **1** is associated with a surprisingly high and those in **2** with unexpectedly low electron densities in the bcps, despite the comparable I⋯N distances. An explanation will be offered in the following section.

### Inter­pretation of very short I⋯*D* (*D* = N and O) contacts   

Before we attempt to inter­pret the results of our experimental electron-density determinations for **1** and **2**, we recall several essential differences between Cl⋯Cl and I⋯*D* (*D* = N and O) halogen bonds. In the latter contacts, iodine is the clearly less electronegative (IUPAC, 1997 ▸) and by far the larger (Cordero *et al.*, 2008 ▸) partner. Bcps are usually located more closely to the less electronegative atom of an inter­action (Gillespie & Popelier, 2001 ▸). As a result, the bcp of such an asymmetric I⋯*D* inter­action falls in the region of the charge depletion next to the larger and less electronegative partner iodine. In contrast to intuition, shorter I⋯*D* contacts may be associated with lower electron density in their bcp, and therefore this criterion does not necessarily qualify as a reliable tool to gauge the strength of a halogen bond. We recall the results of Bianchi *et al.* (2003 ▸, 2004 ▸) mentioned in the preceding section and we will come back to this aspect below. An unexpected trend for different structure models underlines the anti­correlation between electron density in the bcp of an asymmetric I⋯*D* halogen bond and the charge depletion on the I atom. The electron density in the bcp between partners of comparable atomic radius and electronegativity will usually come out higher for an advanced multipole model (MM) than for the conventional independent atom model (IAM); generally speaking, the latter does not qualify for modelling bonding electrons. This expected trend is encountered for covalent bonds between C, N and O atoms and also for the Cl⋯Cl inter­halogen contacts discussed in §2.1. The opposite tendency may be observed for I⋯*D* contacts with a very pronounced σ hole: our cocrystal **2** provides an example for this behaviour. Stepwise expansion of the structure model from the IAM to higher multipoles emphasizes the σ hole and concomitantly leads to a continuous decrease of the electron density in the bcp of the halogen bond; the corresponding compilation of bond critical properties as a function of the multipole expansion is provided in §5 of the supporting information.

**Figure 5:**
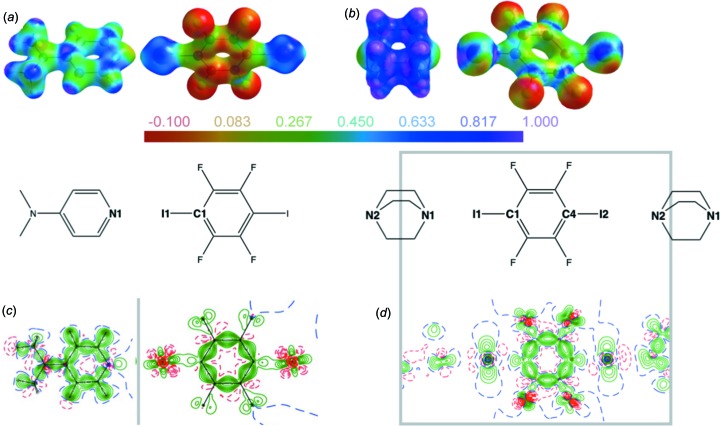
(*a*)/(*b*) Electrostatic potential mapped on an isosurface of electron density ρ = 0.5 e Å^−3^ (*MoleCoolQt*; Hübschle & Dittrich, 2011 ▸) and (*c*)/(*d*) deformation density (contour lines are drawn at 0.1 e Å^−3^) for **1** and **2**. In (*c*), the DMAP and TFDIB molecules are not completely coplanar; the grey line marks their inter­section. In (*d*), the grey box denotes the part of the chemical diagram for which the deformation density has been depicted.

Fig. 5 ▸ visually compares the electrostatic potential (ESP) and the deformation density in our cocrystals **1** (Wang *et al.*, 2018*a* ▸) and **2** (this work), and allows for a discussion of the differences in their I⋯N bonds, despite their apparent chemical and geometric similarities.

The ESP for **2** (Fig. 5 ▸ *b*) shows a distinct positive region (colour coded in magenta) on the I atom, opposite to its bond to carbon – the σ hole! The neighbouring N atom approaches the heavy halogen with a much more negative region (green), thus underlining the strong electrostatic contribution to the halogen bond. Despite the similar I⋯N distance, a σ hole can hardly be perceived for **1** (Fig. 5 ▸ *a*): neither the shape of the isosurface at the I atom nor the colour-coded potential show the clear features observed for **2**. Obvious common features of the ESPs for both compounds are the negative values for F and the positive values for H atoms. We note another difference between **1** and **2**: both the electron density, coded by the shape of the isosurface, and the colour-coded ESP for the I atoms in **1** indicate a rather balanced bonding situation towards its smaller neighbours C and N, contrary to what one might expect for an I atom engaged in a covalent bond and a short contact. We will come back to this aspect below. The deformation densities in Fig. 5 ▸ (bottom) emphasize the differences between **1** and **2**, and Table 1 ▸ summarizes the numerical results.

**Figure 6:**
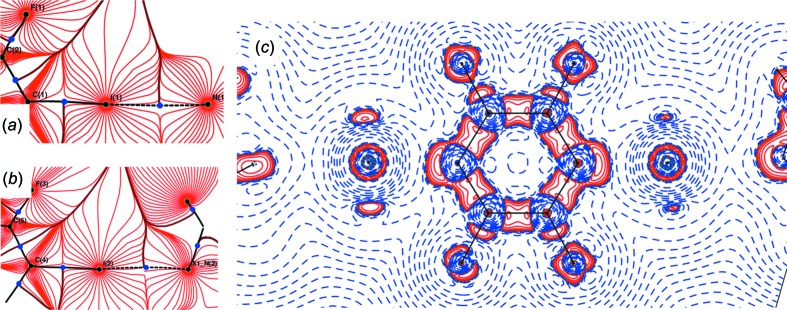
Gradient vector field of the electron density for (*a*) I1⋯N1 and (*b*) I2⋯N2^i^ in **2**; bond paths are shown as black lines and bcps as dark-blue solid circles. (*c*) Laplacian of the electron density for the TFDIB molecule in **2**, with positive values in blue, negative values in red and contours at ±2^*n*^ × 10^−3^ e Å^−5^ (0 ≤ *n* ≤ 20).

A surprisingly high electron density is found in the bcp of the short I⋯N contact in **1** (Table 1 ▸). As expected for a strong inter­action which is more reliably described by an aspherical model, its value increases from 0.257 (5) e Å^−3^ in the IAM to 0.359 (5) e Å^−3^ in the MM. We encountered comparable electron densities in coordinative bonds between N atoms and metal cations (Wang *et al.*, 2009 ▸, 2012 ▸). Similar to the ESP for **1** (Fig. 5 ▸ *a*), its deformation density in Fig. 5 ▸(*c*) shows a rather ‘symmetric’ environment for the I atom, with clearly visible polarization of both smaller neighbouring C and N atoms towards the heavy halogen. From this point of view, the short contact between TFDIB iodine and DMAP nitro­gen is more reminiscent of a three-centre–four-electron bond than of a σ-hole inter­action. In contrast, the I atoms in **2** exhibit the expected charge depletions (Fig. 5 ▸ *d* and Table 1 ▸) in the direction of their close N-atom neighbours. Fig. 5 ▸(*d*) shows zero contour levels (dashed blue lines) in the deformation density of **2**. The bcps between I and N fall in the negative region – the deformation density picture indicates a low electron density in these points, even without resorting to the Laplacian! The σ-hole geometry becomes more visible in the MM: the I⋯N bcps move towards the heavy halogen and their electron density decreases when passing from the IAM to the MM. The pronounced charge depletion associated with the very short I⋯N contact in **2** and the ratio of the atomic radii discussed above leads to electron densities in the bcps of the halogen bonds which are smaller than in the case of the longer I⋯N or I⋯O separations discussed in §2.2. For one of the two symmetrically independent short contacts [I2⋯N2^i^; symmetry code: (i) *x* − 2, *y* − 1, *z*] in **2** the bond path is significantly longer than the inter­atomic distance (Table 1 ▸), and the associated bcp could not be located routinely. More detailed information is given in the *Experimental* section and in the supporting information. The gradient vector plots and the Laplacian of the electron density depicted in Fig. 6 ▸ confirm the presence of a σ hole on both I atoms in **2**.

How do the results of the single-point calculations in Table 1 ▸ compare to the experimentally derived electron density? The covalent C—I bonds are satisfactorily reproduced but the unusual I⋯N inter­actions can be expected to be challenges for theory. Indeed, neither the very strong and more ‘symmetric’ I⋯N contacts in **1** nor the very short halogen bonds in **2** are well described: electron densities in the bcps of the former are underestimated and of the latter are overestimated!

**Figure 7:**
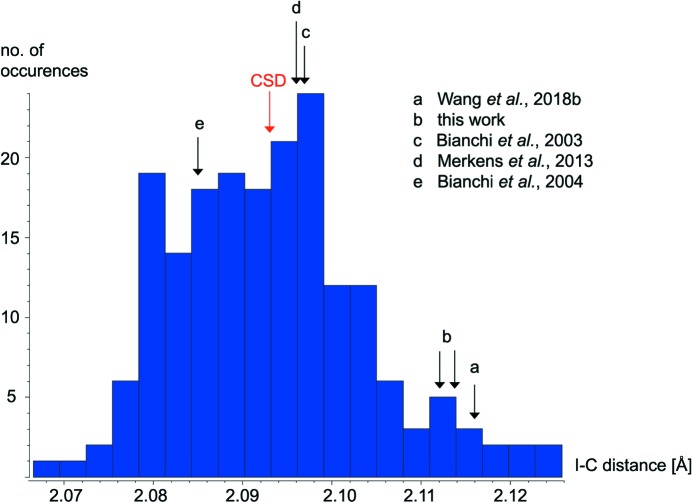
Histogram of I—C distances from TFDIB structures in the CSD (Groom *et al.*, 2016 ▸; error-free structures, no disorder, *T* ≤ 150 K). Selected data for TFDIB cocrystals with short C—I⋯*D* contacts have been included (see text). (For ‘a–d’, *D* = N and for ‘e’, *D* = O; CSD indicates the CSD average.)

High-resolution X-ray diffraction experiments followed by analysis of the derived electron density can provide a very reliable insight into bonding but is, of course, not always feasible. Fortunately, we may offer a geometry-based criterion, which at least for these TFDIB derivatives may help in the inter­pretation and which we first discovered in our detailed analysis of **1**. We recalled the above statement ‘more reminiscent of a three-centre–four-electron bond’: a stronger I⋯*D* inter­action implies weakening of the σ bond and a longer I—C distance. Fig. 7 ▸ shows that this effect is indeed observed and significant.

Both **1** and **2**, despite their very different characteristics in the short inter­molecular I⋯N contacts, show very long and presumably weak I—C bonds opposite to the short contacts. In contrast, the I⋯*D* halogen bonds in the remaining compounds investigated by Bianchi *et al.* [‘c’ in Fig. 7 ▸ (Bianchi *et al.*, 2003 ▸) and ‘e’ in Fig. 7 ▸ (Bianchi *et al.*, 2004 ▸)] and our group (‘d’ in Fig. 7 ▸; Merkens *et al.*, 2013 ▸) are associated with unexceptional I—C distances, close to the database average. It is tempting to search the CSD for a more general anti­correlation between short I⋯*D* contacts and long I—C bonds but the result is less conclusive than one might intuitively expect. Halogen bonds with their strong electrostatic contribution cover a wide range of distances; contacts between iodine and a small electronegative nucleophile, such as nitro­gen or oxygen, may be as short as in **1**, *i.e.* in the range of 2.6 Å. The upper limit is largely a matter of taste but will often be associated with the sum of the van der Waals radii and will at least extend to 3.3 or 3.4 Å. The covalent bond between C and I in the TFDIB molecule is largely dominated by orbital overlap and ranges between 2.07 and 2.12 Å. In either case, a realistic error bar for structures derived from diffraction data at standard resolution is about equally high and amounts to *ca* 0.01 Å. The earlier database entries for our compound **2** (CSD refcodes ISIHUN (Bolte, 2004 ▸) and ISIHUN01 (Cinčić *et al.*, 2008 ▸)] provide an instructive proof for this statement: two low-temperature data collections (100 and 180 K) and refinements were conducted independently and resulted in I—C bond lengths between 2.110 and 2.121 Å. In summary, correlation between a first variable of *ca* 0.7±0.01 Å and a second of *ca* 0.05±0.01 Å is attempted, and the result is necessarily noisy. The few charge–density studies with their obviously higher resolutions can, of course, be expected to be more precise than the overall database screening. A (still rather poor) anti­correlation can be perceived when the search is limited to the strongest halogen bonds (I⋯N < 2.8 Å) for which a significant effect on I—C can be expected. The corresponding scatterplot is available in the supporting information.

The difference between **1** and **2** is also reflected in the electron densities in the I—C bcps (Table 1 ▸). In **1**, ρ_bcp_ for I⋯N and I—C show the same trend and increase, as is to be expected, when the IAM is replaced by the aspherical MM. In contrast, the values for ρ_bcp_ of I—C in **2** are hardly affected by the model and are in close agreement with earlier results (Bianchi *et al.*, 2003 ▸, 2004 ▸; Merkens *et al.*, 2013 ▸).

### Energy density considerations   

In addition to the electron density ρ and its Laplacian, energy densities have been used to categorize secondary inter­actions. The kinetic energy density *G* and the ratio between kinetic energy density and electron density, *G*/ρ in the bcp, were derived as suggested by Abramov (1997 ▸), and the potential energy density *V* was obtained according to the local virial theorem (Espinosa *et al.*, 1998 ▸, 1999 ▸). *G*/ρ has proven useful for classifying hydrogen bonds (Şerb *et al.*, 2011 ▸) but does not represent a very sensitive qualifier for halogen bonds. More successful was an alternative criterion: the total energy density *E*, the difference between the (positive) kinetic energy density *G* and the (negative) potential energy density *V*, assumes negative values for covalent bonds (Cremer & Kraka, 1984 ▸) and only for the shortest Cl⋯Cl inter­actions (Wang *et al.*, 2017 ▸). In line with this argument, an unambiguously negative value for *E* is calculated for the short I⋯N contact in **1**. Espinosa *et al.* (2002 ▸) have suggested the ratio |*V*|/*G* to distinguish between pure closed-shell and incipient shared-shell inter­actions. With respect to this criterion, the short inter­molecular contact in **1** is characterized by |*V*|/*G* = 1.42 (Wang *et al.*, 2018*a* ▸) and falls in the regime of shared inter­actions. When we apply the same qualifiers *E* and |*V*|/*G* to a significantly longer but still relevant halogen bond, *e.g.* the short I⋯O contact in the hypervalent Togni reagent (Wang *et al.*, 2018*b* ▸), a slightly positive total energy density *E* and |*V*|/*G* = 0.86 are obtained, indicating a closed-shell inter­action as expected. In summary, both criteria are promising and distinguish between presumably incipient shared- and closed-shell inter­actions. Our compound **2**, with its unexpectedly low electron density in the bcps of the short I⋯N contacts, does remain ambiguous, again, with respect to these criteria. When we focus on experiment, the total energy densities *E* are close to 0, similar to what is seen for much longer I⋯*D* contacts; the ratio |*V*|/*G* adopts values close to 1.0 (Table 2 ▸), *i.e.* in-between closed and shared inter­actions.

**Figure 8:**
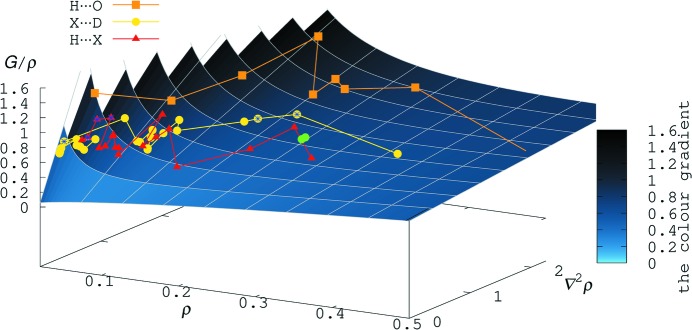
Ratio *G*/ρ as a function of the electron density ρ and its Laplacian; all qu­anti­ties refer to the bcp. Experimental results for halogen bonds are shown in yellow, for H⋯*X* in red and for H⋯O in orange. The yellow circles marked with an asterisk (*) represent the experimental and the green circles the theoretically calculated values for **2**.

The results of the single-point calculation suggest a different inter­pretation, with significantly negative values for *E* and |*V*|/*G* = 1.20; both qualifiers seem to indicate a more shared inter­action. As a result of the very pronounced σ hole in close vicinity to the I⋯N bcp in **2**, ρ_bcp_ in these contacts is much lower than expected and properties derived from the electron density are equally affected. We have summarized different properties in the bcps for halogen bonds and other classes of intermolecular contacts in Fig. 8 ▸. This graph was originally suggested based on data compiled in our 2017 article (Wang *et al.*, 2017 ▸) and later extended (Wang *et al.*, 2018*a* ▸) to cover the short I⋯N contact in **1**; it relates relates *G*/ρ with the electron density ρ and its Laplacian ∇^2^. High values for *G*/ρ are only observed for hydrogen bonds to oxygen (orange-coloured data points in Fig. 8 ▸). Halogen bonds (yellow data points) are less sensitive with respect to this qualifier and better categorized with the help of *E* or |*V*|/*G*; they do cover, however, an impressive range of electron density in their (3,−1) critical points. In **2** and, most likely, in related compounds with very short σ-hole contacts, every classification exclusively based on bcp properties is associated with a large uncertainty: the discrepancy between experimentally derived [yellow circles marked with an asterisk (*)] and theoretically calculated (green circles) qualifiers is reflected in the different location of the highlighted data points in Fig. 8 ▸.

### Raman spectroscopy as a diagnostic tool for short halogen bonds   

**Figure 9:**
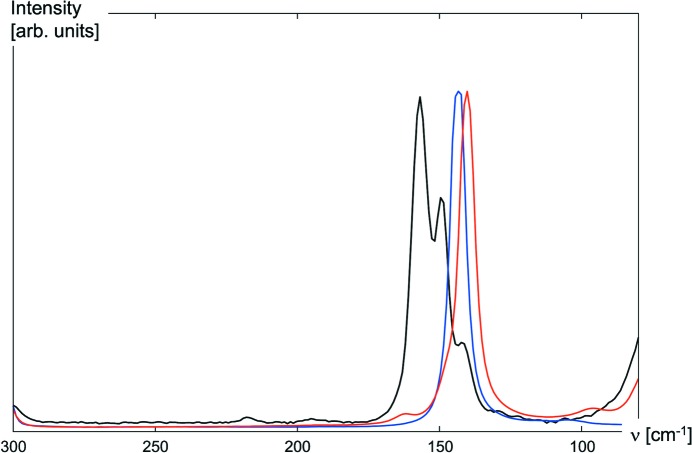
Raman spectra for TFDIB (black) and its cocrystals **1** (red) and **2** (blue) in the frequency range 300–80 cm^−1^.

Diffraction data at standard resolution qualify for discussions of crystal engineering, being sufficient to obtain atomic coordinates and derive inter­atomic distances and angles. Considerations concerning the electron density and the nature of bonds and short contacts require high-resolution data. In the preceding sections, we suggested a link between halogen-bond strength and (precise) mol­ecular geometry. So far, our discussion about halogen bonding has relied on the results of diffraction experiments. Based on the results for TFDIB and its cocrystals **1** and **2**, we suggest Raman spectroscopy as a straightforward alternative tool to probe short inter­molecular I⋯N contacts. On the one hand, intense Raman signals may be expected for modes to which the highly polarizable I atoms contribute. On the other hand, the results discussed in §2.3 suggest that short I⋯N inter­actions lead to longer and presumably weaker I—C bonds, and a shift of the corresponding absorption to lower frequencies may be perceived as an indicator for halogen bonds. Fig. 9 ▸ shows the Raman spectra for TFDIB and its cocrystals **1** and **2** in the frequency range in which we expect C—I vibrations.

Our inter­pretation of the observed red shift in the presence of I⋯N halogen bonds relies on the good agreement between observed and calculated spectra compiled in Table 3 ▸.

**Figure 10:**
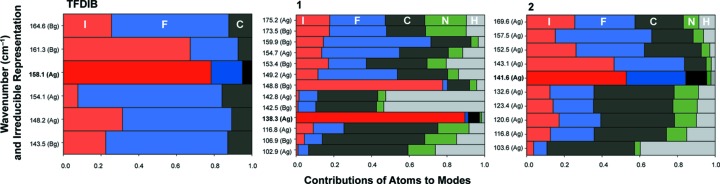
Contributions of atoms to Raman-active phonon modes in the range from 100 to 200 cm^−1^ for TFDIB and cocrystals **1** and **2**. The modes with the most significant contribution of iodine in this range are highlighted in bold for the labels and more intense colours for the bar chart. These highlighted wavenumbers have been included in Table 3 ▸ and closely match the experimental values depicted in Fig. 9 ▸.

The results of the phonon calculations in the range of the C—I bonds are summarized in Fig. 10 ▸.

Furthermore, the results from the Raman spectra are in good agreement with results from bonding analysis. Integrated Crystal Orbital Hamilton Population (ICOHP) values have been used successfully to evaluate the strengths of hydrogen and tetrel bonds within crystal structures (Deringer *et al.*, 2014 ▸, 2017 ▸; George & Dronskowski, 2017 ▸). The ICOHP for the I⋯N inter­action in the cocrystals **1** and **2**, and for the C—I bond in all three compounds TFDIB, **1** and **2** have been included in Table 3 ▸ and match the red shift in the Raman spectra. ICOHP values for I—C are significantly smaller for the cocrystals and indicate weakening of the covalent I—C bond as a result of the short halogen bonds in the cocrystals.

We already mentioned that Karadakov, Bruce and co-workers (Roper *et al.*, 2010 ▸) could synthesize a series of halo­gen-bonded cocrystals by grinding. In this light of mechanochemistry, Raman spectroscopy as an alternative tool to analyse halogen bonding without the necessity of crystallization and single-crystal diffraction gains significant importance.

## Experimental   

### Synthesis and crystallization   

High-quality single crystals of **2** were obtained from an equimolar solution of the components as first described by Cinčić *et al.* (2008 ▸); matching elemental analysis and powder patterns were obtained. Cocrystal **2** may also be obtained mechanically by grinding the constituents (Cinčić *et al.*, 2008 ▸); even when the constituents are only mixed as solid powders without grinding, partial formation of cocrystalline **2** is observed. The corresponding powder patterns are provided in the supporting information.

### Single-crystal X-ray diffraction   

Intensity data for **2** were collected at 100 K on a Stoe Stadivari goniometer equipped with a Dectris Pilatus 200K detector using Mo *K*α radiation (λ = 0.71073 Å). The radiation source was a XENOCS microsource equipped with multilayer optics. An Oxford Cryosystems 700 controller was used to ensure temperature stability during data collection. The intensity data were processed with *X-AREA* (Stoe & Cie, 2017 ▸). Direction-dependent scaling in the subprogram *LANA* and its relationship to the well-established *SORTAV* program (Blessing, 1995 ▸) have been described by Koziskova *et al.* (2016 ▸). Crystal data and information concerning data collection are compiled in Table 4 ▸. The independent atom refinement was performed by full-matrix least squares on *F* ^2^ (Sheldrick, 2015 ▸). H atoms were treated as riding, with C—H = 0.99 Å and *U*_iso_(H) = 1.2*U*_eq_(C).

### Multipole refinement and AIM analysis   

The final IAM served as the starting point for the multipole model (MM). Equivalent reflections were averaged with the help of the program *MERGEHKLF5* (Schreurs, 2004 ▸). Multipole refinements on *F* ^2^ based on the Hansen–Coppens formalism for aspheric atomic density expansion (Hansen & Coppens, 1978 ▸) were carried out with the program *XD2006*; the VM data bank based on unpublished work by Volkov and Macchi was used (Volkov *et al.*, 2006 ▸). Refinement was conducted with all intensity data. The refined anisotropic displacement parameters were in agreement with the rigid bond postulate (Hirshfeld, 1976 ▸). Refinement of anharmonic displacement parameters, more specifically third- and fourth-order Gram–Charlier coefficients for iodine and third-order coefficients for F atoms (Sørensen *et al.*, 2003 ▸), was attempted but not further pursued because the resulting structure models were associated with slightly more favourable agreement factors but higher residual electron densities at the expense of significantly more refined variables. The final successful multipole refinement converged for low and symmetric residual electron-density maxima and minima (Table 4 ▸, and Fig. S12 in the supporting information). The remaining features in the final difference Fourier map are located close to the I atom and indicate limitations of the atom-centred multipole model restricted to the valence shell rather than anharmonic motion or inconsistencies with the intensity data. This inter­pretation is corroborated by the fractal dimension, probability distribution histograms and normal probability plots provided in the supporting information (Figs. S9–S11). The final MM comprised multipoles up to hexa­deca­poles for non-H atoms and up to bond-directed dipoles for the H atoms. The space group did not require any symmetry constraint on multipoles; chemical constraints were introduced for C and H atoms, *i.e.* all methyl­ene C atoms, the I-substituted C atoms in the TFDIB molecule and the F-substituted C atoms in the TFDIB molecule, and all H atoms in the DABCO molecule were treated as chemically equivalent (Table S4 in the supporting information). Contraction parameters κ for non-H atoms were refined freely; κ for H was constrained to 1.2 and κ′ for all atoms were kept unrefined at the default values of 1.0 for non-H and 1.2 for H atoms. In the MM, C—H distances were constrained to 1.09 Å. The topology of the experimental electron density was analyzed according to Bader’s AIM theory (Bader, 1990 ▸); the search for critical points was con­ducted with *XDPROP* (as supplied with *XD2006*; Volkov *et al.*, 2006 ▸) and *TOPXD* (Volkov *et al.*, 2000 ▸). For one of the short contacts in **2** [I2⋯N2^i^; symmetry code: (i) *x* − 2, *y* − 1, *z*], both programs failed to locate the bcp by the usual approach based on short contacts between atom pairs (§5 of the supporting information), although a graphical inter­polation indicated relevant electron density along the inter­atomic path. A search in the asymmetric unit of **2** with *TOPXD* was successful and gave the same ρ_bcp_ as the graphical estimate.

### Raman spectroscopy   

Raman spectra were obtained with a Horiba LABRAM HR instrument equipped with a 633 nm HeNe excitation laser.

### Computational methods   

In order to complement the experimental electron-density results with a theoretical description of the strongest inter­molecular inter­actions, calculations were performed on a three-molecule DMAP–TFDIB–DMAP aggregate in the case of **1** and on a four-mol­ecule TFDIB–DABCO–TFDIB–DABCO aggregate in the case of **2**. The experimentally observed geometries were used for single-point calculations, which were performed with *GAUSSIAN09* (Frisch *et al.*, 2009 ▸) at the density functional theory (DFT) level with the B3LYP functional (Becke, 1993 ▸; Lee *et al.*, 1998 ▸; Vosko *et al.*, 1980 ▸; Stephens *et al.*, 1994 ▸) and the MIDIX basis set (Thompson *et al.*, 2001 ▸). The wavefunctions obtained through these calculations were used for the topological analysis of the resulting electron density with the help of the program *AIMAll* (Keith, 2017 ▸) Figs. S16 and S17 in the supporting information show these results for **2**, the compound for which the experimental electron density is reported for the first time in this work.

Forces for all phonon calculations and the preceding structural optimizations were calculated with dispersion-cor­rected DFT as implemented in *VASP* (Kresse & Hafner, 1993 ▸, 1994 ▸; Kresse & Furthmüller, 1996 ▸), with strict convergence criteria of Δ*E* < 10^−7^ (10^−5^) eV per cell for electronic (structural) optimizations, respectively. In contrast to the aforementioned calculations based on mol­ecular aggregates, periodic boundary conditions were used for the calculations. We used the projector augmented-wave method (Blöchl, 1994 ▸; Kresse & Joubert, 1999 ▸), with a plane wave cut-off of 500 eV, and the Perdew–Burke–Ernzerhof (PBE) functional (Perdew *et al.*, 1996 ▸). In addition to the PBE functional, we also used the ‘D3’ correction of Grimme and co-workers, together with Becke–Johnson damping (Grimme *et al.*, 2010 ▸, 2011 ▸). Instead of the traditional damping parameters, as fitted by Grimme and co-workers, we used those suggested by the group of Sherrill (*s*_6_ = 1.00, *s*_8_ = 0.358940, α_1_ = 0.012092 and α_2_ = 5.938951) (Smith *et al.*, 2016 ▸). In previous work, this method was successfully applied to describe the thermal expansion of the halogen-bond-containing compound penta­chloro­pyridine (George *et al.*, 2017 ▸). The prediction of thermal expansion with the help of the quasi-harmonic approximation relies very much on a good description of the underlying frequencies calculated within the harmonic approximation.

To perform the phonon calculations, we used the finite displacement method as implemented in *Phonopy* (https://atztogo.github.io/phonopy/), with a displacement of 0.01 Å and a 3 × 2 × 4 supercell for TFDIB, a 3 × 2 × 2 supercell for TFDIB·DABCO, **1**, and a 2 × 1 × 2 supercell for TFDIB·DMAP, **2** (Togo *et al.*, 2008 ▸; Togo & Tanaka, 2015 ▸). Furthermore, the force calculations were performed at the Γ-point. The irreducible representations of the phonon modes at the Γ-point were also determined with the help of *Phonopy*. The atomic contributions to each phonon mode were calculated and visualized with the help of *AtomicContributions* (Version 1.3; George, 2019 ▸).

The ICOHP values were calculated for the optimized structures with the help of *Lobster* (Version 3.1.0; Dronskowski & Blöchl, 1993 ▸; Deringer *et al.*, 2011 ▸; Maintz *et al.*, 2013 ▸, 2016 ▸). The following basis functions of the pbeVasp­Fit2015 basis set were used: 1*s* for H, 2*s* 2*p* for C, N, and F, and 5*s* and 5*p* for I. For all three compounds, the charge spilling was below 1.5%, which indicates a very reliable projection.

## Conclusions   

Experimental electron-density studies on compounds with short inter­molecular Cl⋯Cl contacts are in agreement with the commonly accepted σ-hole theory. The nucleophile, the electrophile with the σ hole and its covalently bonded partner atom giving rise to this positive region are arranged in a linear fashion. Longer Cl⋯Cl distances are associated with low electron density in the bcp; only little, if any, information about the nature and strength of the inter­action can be extracted. Short Cl⋯Cl contacts are associated with clear features in the electron density and derived properties, such as the Laplacian or the ESP: the charge depletion on the electrophile and the polarization of the nucleophile may be perceived, and the electron density in the bcp of the inter­chlorine contact increases for shorter distances.

For inter­actions between I atoms and small electronegative partners *D* (such as N or O), only a few charge–density studies have been conducted. The small number of experimental observations does not allow a simple trend to be established for the electron density in the bcp as a function of inter­atomic distance but rather suggests that three cases can be distinguished. (*a*) Our compound **1**, with its very short I⋯N inter­actions of less than 2.7 Å, shows a rather symmetric electronic situation of the heavy halogen and resembles a three-centre–four-electron bond. The MM description suggests a higher electron density in the bcp of the I⋯*D* inter­action than the IAM. The absolute value of electron density in the bcp exceeds that of published halogen bonds and is similar to coordinative bonds between a large cation and a small nucleophile. (*b*) Significantly longer and presumably weaker halogen bonds, with I⋯*D* separations of about 3 Å, reflect the commonly accepted σ-hole features. The electron density in their bcp is scarcely affected by details of the structure model. (*c*) Our compound **2** represents the first example in-between the above-mentioned categories for which the electron density has been established experimentally. Although it does not differ much from case (*a*) with respect to the I⋯*D* contact distance, **2** is clearly asymmetric with respect to the electron density about the heavy halogen. It still represents a very short halogen bond and its ESP matches the requirements for an electrostatically favourable inter­action, but due to its vicinity to the charge depletion, the electron density in the associated bcp is rather low. More generally, we expect an analogous effect for related halogen-bonded compounds in which ρ_bcp_ may be reduced due to its proximity to the σ hole. With this surprising feature, we add yet a different flavour to halogen bonding! Although only a few experimental electron-density studies involving halogen bonds with iodine are available, we are tempted to make a comparison with hydrogen bonds, an inter­action for which a wealth of high-resolution diffraction data exists. Based on a large number of H⋯*X* (*X* = H, C, N, O, F, S, Cl and π) contacts, Espinosa and co-workers (Mata *et al.*, 2010 ▸) have detected that increasing electronegativity of the acceptor *X* leads to a more extended range of inter­actions of an entirely closed-shell nature. In agreement with these findings, all Cl⋯Cl contacts compiled in Fig. 3 ▸ follow the same trend; in contrast, very short inter­actions involving the less electronegative iodine are borderline cases with shared inter­actions. Additional experimental data on short halogen bonds between iodine and small nucleophiles will be required to confirm or disprove this analogy.

## Supplementary Material

Crystal structure: contains datablock(s) global, I. DOI: 10.1107/S205322961901132X/rh3011sup1.cif

Structure factors: contains datablock(s) I. DOI: 10.1107/S205322961901132X/rh3011Isup2.hkl

Supporting information concerning data quality, completeness, convergence and residual electron density. DOI: 10.1107/S205322961901132X/rh3011sup3.pdf

Additional CIF reporting the multipole refinement. DOI: 10.1107/S205322961901132X/rh3011sup4.txt

CCDC reference: 1946856

## Figures and Tables

**Table 1:** Properties of the electron density in the bcps of the I⋯N contacts and I—C bonds in **1** and **2** *R*_12_ is the bond path, *d*_1_ and *d*_2_ its components, ρ the electron density and ∇^2^ the Laplacian in the bcp. Results labelled as ‘calc’ were obtained from single-point calculations in experimentally established MM geometry.

Compound	Bond	Model	Distance	*R*_12_	*d*_1_	*d*_2_	ρ	∇^2^ρ
			(Å)	(Å)	(Å)	(Å)	(e Å^−3^)	(e Å^−5^)
**1**	I1⋯N1	MM	2.6622 (4)	2.6625	1.4274	1.2351	0.359 (5)	1.95 (2)
		calc		2.6629	1.3819	1.2810	0.250	1.90
		IAM	2.6630 (6)	2.6628	1.4864	1.1764	0.257 (5)	2.29 (2)
	I1—C1	MM	2.1168 (4)	2.1190	1.1649	0.9541	0.85 (3)	2.23 (6)
		calc		2.1168	1.0828	1.0340	0.81	1.06
		IAM	2.1176 (4)	2.1181	1.1862	0.9319	0.69 (3)	3.17 (6)
								
**2**	I1⋯N1	MM	2.7374 (11)	2.7616	1.4660	1.2956	0.19 (2)	2.071 (5)
		calc		2.7374	1.4144	1.3230	0.229	1.716
		IAM	2.7350 (9)	2.7351	1.5253	1.2097	0.230 (2)	2.067 (6)
	I1—C1	MM	2.1134 (10)	2.1147	1.1300	0.9847	0.69 (3)	4.72 (5)
		calc		2.1136	1.0870	1.0266	0.79	0.91
		IAM	2.1150 (10)	2.1130	1.1834	0.9296	0.70 (2)	3.18 (8)
	I2⋯N2^i^	MM	2.7453 (11)	2.8461	1.5145	1.3316	0.16 (2)	1.807 (5)
		calc		2.7453	1.4158	1.3295	0.228	1.668
		IAM	2.7457 (10)	2.7544	1.5299	1.2140	0.227 (2)	2.054 (6)
	I2—C4	MM	2.1119 (10)	2.1200	1.1391	0.9809	0.69 (3)	4.61 (4)
		calc		2.1146	1.0827	1.0319	0.78	1.01
		IAM	2.1134 (10)	2.1132	1.1835	0.9297	0.70 (2)	3.18 (8)

Symmetry code: (i) *x* − 2, *y* − 1, *z*.

**Table 2:** Properties of the electron density in the bcps of the inter­molecular contacts in **2** *R*_12_ is the bond path, *d*_1_ its component with respect to the first atom, ρ the electron density, ∇^2^ the Laplacian in the bcp, *G* the kinetic, *V* the potential and *E* the total energy density. Results labelled as ‘calc’ were obtained from single-point calculations in experimentally established MM geometry.

Bond	Distance	*R*_12_	*d*_1_	ρ	∇^2^	*G*	*G*/ρ	*V*	|*V*|/*G*	*E*
	(Å)	(Å)	(Å)	(e Å^−3^)	(e Å^−5^)	(a.u.)	(a.u.)	(a.u.)		(a.u.)
I1⋯N1	2.7374 (11)	2.7616	1.4660	0.19 (2)	2.071 (5)	0.0216	0.78	−0.0217	1.00	−0.0001
calc		2.7374	1.4144	0.229	1.716	0.0222	0.65	−0.0267	1.20	−0.0045
I2⋯N2^i^	2.7453 (11)	2.8461	1.5145	0.16 (2)	1.807 (5)	0.0181	0.77	−0.0174	0.96	0.0007
calc		2.7453	1.4158	0.228	1.668	0.00217	0.64	−0.0261	1.20	−0.0044
										
F1⋯H15*A* ^ii^	2.59	2.6176	1.4785	0.038 (2)	0.553 (2)	0.0043	0.77	−0.0029	0.67	0.0014
F2⋯H16*A* ^iii^	2.47	2.4722	1.4911	0.038 (2)	0.708 (2)	0.0054	0.96	−0.0035	0.65	0.0019
F4⋯H12*B* ^iv^	2.41	2.4193	1.4421	0.046 (2)	0.836 (2)	0.0065	0.95	−0.0043	0.66	0.0022
F3⋯F3^v^	2.893 (2)	2.8958	1.4658	0.045 (2)	0.765 (2)	0.0060	0.89	−0.0040	0.67	0.0020

Symmetry codes: (i) *x* − 2, *y* − 1, *z*; (ii) −*x* + 2, −*y* + 1, −*z* + 2; (iii) *x* − 1, *y* − 1, *z*; (iv) −*x* + 2, −*y* + 1, −*z* + 1; (v) −*x*, −*y*, −*z* + 1.

**Table 3:** Weakening of I—C by strong halogen bonds: experimentally observed *versus* calculated Raman frequencies and Integrated Crystal Orbital Hamilton Population (ICOHP) for TFDIB, **1** and **2**

compound	TFDIB	**1**	**2**
ν_exp _ (cm^−1^)	157	140	143
ν_calc_ (cm^−1^)	158	138	142
ICOHP I⋯N (eV)		−1.1	−0.8
ICOHP I–C (eV)	−5.7	−4.8	−4.9

**Table 4:** Experimental details for **2**

Crystal data	
Chemical formula	C_6_H_12_N_2_·C_6_F_4_I_2_
*M*_r_	514.04
Crystal system, space group	Triclinic, *P* 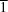
Temperature (K)	100
*a*, *b*, *c* (Å)	6.77971 (9), 10.82624 (17), 11.36217 (17)
α, β, γ (°)	107.3260 (12), 92.9637 (12), 104.7718 (12)
*V* (Å^3^)	762.42 (2)
*Z*	2
Radiation type	Mo *K*α
μ (mm^−1^)	4.16
Crystal size (mm)	0.27 × 0.07 × 0.03

Data collection	
Diffractometer	Stoe & Cie Stadivari goniometer with a Pilatus 200K area detector
Absorption correction	Multi-scan (*X-AREA*; Stoe & Cie, 2017 ▸)
*T*_min_, *T*_max_	0.245, 0.691
No. of measured, independent and observed [*I* > 2σ(*I*)] reflections	143675, 12807, 9751
*R*_int_	0.049
(sin θ/λ)_max_ (Å^−1^)	1.004

Refinement (IAM)	
*R*[*F* ^2^ > 2σ(*F* ^2^)], *wR*(*F* ^2^), *S*	0.018, 0.034, 1.00
No. of reflections	12807
No. of parameters	181
Δρ_max_, Δρ_min_ (e Å^−3^)	0.80, −1.16

Refinement (MM)	
*R*[*F* ^2^ > 2σ(*F* ^2^)], *wR*(*F* ^2^), *S*	0.016, 0.022, 0.99
No. of reflections	12807
No. of parameters	464
Δρ_max_, Δρ_min_ (e Å^−3^)	0.64, −0.64

Computer programs: *X-AREA* (Stoe & Cie, 2017 ▸), *SHELXS97* (Sheldrick, 2008 ▸), *SHELXL2018* (Sheldrick, 2015 ▸) and *PLATON* (Spek, 2009 ▸).
