# Supplementary material for: The many flavours of halogen bonds – message from experimental electron density and Raman spectroscopy
Source: Acta Crystallogr C Struct Chem. 2019 Aug 22;75(Pt 9):1190–201. doi: 10.1107/S205322961901132X (PMC6727171; doi:10.1107/S205322961901132X)
Supplement: Supplementary file 3 [file c-75-01190-sup3.pdf]

STRUCTURAL CHEMISTRY

Volume 75 (2019)

Supporting information for article:

The many flavours of halogen bonds – message from experimental electron density and Raman spectroscopy

Ruimin Wang, Janine George, Shannon Kimberly Potts, Marius Kremer, Richard Dronskowski and Ulli Englert

Supporting Information:

The many flavours of halogen bonds – messages from experimental electron density and Raman spectroscopy

Ruimin Wang, Janine George, Shannon Kimberly Potts, Marius Kremer, Richard Dronskowski and Ulli Englert

August 10, 2019

List of Tables

S1 I···N and C—I distances in TFDIB···N donor adducts for which an experimental electron density is available. . . . 4
S2 Resolution & completeness statistics (cumulative and Friedel pairs averaged) . . . 4
S3 R-value statistics (IAM) as a function of resolution (in resolution shell) 5
S5 Bond distances (Å) for **2**. . . . 7
S6 Bond angles (°) for **2**. . . . 7
S7 Angles (°) of intermolecular interactions for **2**. . . . 7
S8 Topological properties in the bond critical points of the experimental electron density. 15
S4 Contraction parameters and Population coefficients 19

List of Figures

S1 Mechanochemical synthesis of **2**: solids shortly mixed without grinding, black; cocrystals from solution, blue. . . . 3
S2 scatterplot: Anticorrelation between I—C distances and I···N 3
S3 MM refinement results for **2**. . . . 6
S4 Displacement ellipsoid plot of TFDIB DABCO aggregate in **2** at the 90% probability level; hydrogen atoms omitted. . . . 7
S5 DRplot for **2**. . . . 8
S6 Scatterplot F^2_{obs}/F^2_{calc} vs $sin\theta/\lambda$ for X-ray refinement result (MM) for **2**. 9
S7 Averaged $K = \sum(F^2_{obs})/\sum(F^2_{calc})$ vs $sin\theta/\lambda$ for X-ray refinement result (MM) for **2**. 9
S8 Averaged $K = \sum(|F_{obs}|)/\sum(|F_{calc}|)$ vs $sin\theta/\lambda$ for X-ray refinement result (MM) for **2**. . . . 9
S9 Fractal dimension plots [1, 2] vs residual electron density. . . . 10
S10 Probability distribution histogram [1, 2] vs residual electron density. . . . 10
S11 Normal probability plots [1, 2]. . . . 11

Institute of Inorganic Chemistry, RWTH Aachen University, Landoltweg 1, Aachen 52074, Germany. Fax: +49 241 8092 288; Tel: +49 241 809 4666 ; E-mail: ullrich.englert@ac.rwth-aachen.de

S12 Residual electron density after the multipole refinement. Green: positive red: negative blue: zero 11
S13 xdprop bcp part results for **2**. 12
S14 I2$\cdots$N2^i electron density ρ (e/Å^3) distribution for **2**. 14
S15 Short N$\cdots$I contact in the aggregate in **2**; Hirshfeld surface is shown for the TFDIB and DABCO moiety. The surface colours code the electrostatic potential (red negative, blue positive) [6]. 16
S16 $\mid \nabla\rho \mid$ from calculation; bond paths are shown as black lines, bond critical points as black solid circles, and ring critical points as green solid circles.[7] 17
S17 Laplacian of the electron density obtained by calculation ($\nabla^2\rho$); contours are at $\pm 2^n \cdot 10^{-3}$ a.u. Positive values are in blue and negative values in red **2**. 17

1 Experimental powder patterns for cocrystals grown from powder and by grinding.

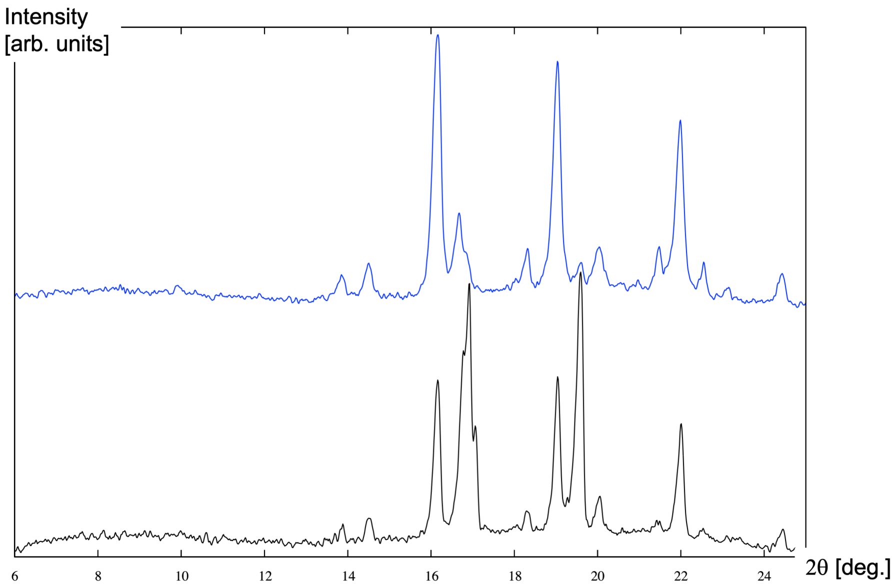

Figure S1: Mechanochemical synthesis of **2**: solids shortly mixed without grinding, black; cocrystals from solution, blue.

2 scatterplot: Anticorrelation between I—C distances and I$\cdots$N

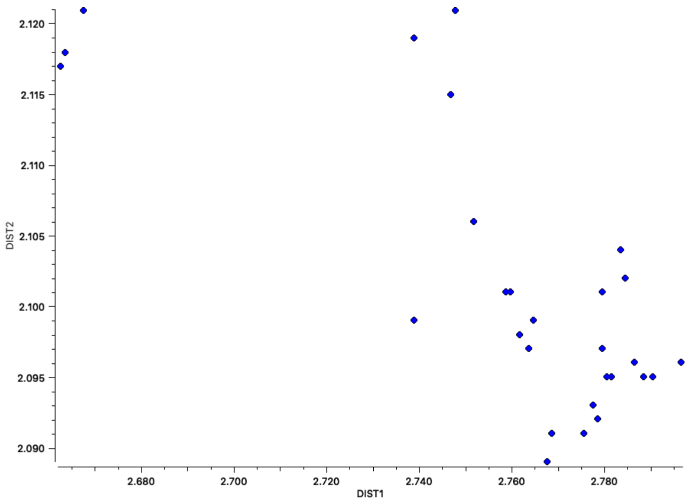

Figure S2: scatterplot: Anticorrelation between I—C distances and I$\cdots$N for 29 error-free CSD entries of TFDIB cocrystals with I$\cdots$N less or equal 2.8 Å(error-free structures without disorder, powder structures excluded). The correlation coefficient amounts to –0.764.

Table S1: I···N and C—I distances in TFDIB···N donor adducts for which an experimental electron density is available.

Compound	Reference	I···N [Å]	C–I [Å]	ρ [eÅ^{-3}]
BPE·TFDIB	Bianchi *et al.*, 2003	2.7804(8)	2.0969(7)	0.236(2)
Al(acacCN)$_3$·TFDIB	Merkens *et al.*, 2013	2.833(3)	2.096(2)	0.154(12)
**1**	Wang *et al.*, 2018b	2.6622(4)	2.1168(4)	0.359(4)
**2**	this work	2.7374(11)	2.1134(10)	0.19(2)
		2.7453(11)	2.1119(10)	0.16(2)

3 Data collection details

Temperature (K)	100
Radiation (Å)	MoK_α 0.71073
θ Min–Max (°)	3.1–45.50
Dataset	-13: 11 ; -21: 19 ; -22: 22
Tot., Uniq. Data, R(int)	143675, 12807, 0.0493

Table S2: Resolution & completeness statistics (cumulative and Friedel pairs averaged)

```
Theta sin(th)/Lambda Complete  Expected Measured  Missing
----------------------------------------------------------
 20.82     0.500     0.996         1595     1589        6
 23.01     0.550     0.997         2127     2121        6
 25.24     0.600     0.998         2764     2758        6
 27.51     0.650     0.998         3514     3508        6
 29.84     0.700     0.998         4386     4377        9
 32.21     0.750     0.996         5386     5367       19
 34.65     0.800     0.996         6525     6500       25
 37.17     0.850     0.995         7849     7809       40
 39.77     0.900     0.994         9316     9258       58
 42.47     0.950     0.992        10938    10853       85
 45.29     1.000     0.991        12800    12683      117
 45.50     1.004     0.990        12930    12807      123

Note: The Reported Completeness refers to the Actual H,K,L
Index Range
```

4 Structure refinement

Table S3: R-value statistics (IAM) as a function of resolution (in resolution shell)

Theta	sin(Th)/L	#	R1	wR2	S	Rs	av(I/SigW)	av(I)	av(SigW)
12.38	0.302	343	0.014	0.036	2.360	0.008	58.23	6325.94	82.61
15.68	0.380	349	0.015	0.035	1.997	0.011	50.00	3787.29	58.37
18.02	0.435	351	0.012	0.027	1.582	0.009	50.33	2839.75	39.90
19.90	0.479	364	0.014	0.030	1.630	0.011	46.77	2281.01	34.86
21.51	0.516	333	0.014	0.029	1.326	0.014	37.62	1848.92	33.70
22.94	0.548	364	0.013	0.027	1.197	0.016	35.57	1616.70	31.07
24.22	0.577	345	0.014	0.029	1.180	0.018	32.44	1326.62	28.89
25.40	0.603	353	0.014	0.029	1.037	0.022	28.24	1168.97	29.60
26.49	0.628	355	0.018	0.034	1.058	0.029	23.30	1008.67	31.98
27.52	0.650	351	0.019	0.036	0.949	0.037	19.69	895.69	35.12
28.49	0.671	341	0.021	0.041	1.065	0.037	19.18	894.16	35.04
29.41	0.691	360	0.023	0.045	1.076	0.044	16.79	718.92	33.06
30.28	0.709	340	0.024	0.048	1.036	0.048	15.64	726.70	35.84
31.12	0.727	352	0.022	0.040	0.861	0.052	15.32	595.23	32.18
31.93	0.744	340	0.017	0.033	0.827	0.041	18.05	564.27	24.07
32.71	0.760	349	0.015	0.029	0.715	0.042	17.93	492.42	21.51
33.46	0.776	364	0.017	0.032	0.756	0.044	17.22	490.32	22.27
34.20	0.791	337	0.017	0.036	0.815	0.047	16.81	471.43	22.85
34.91	0.805	337	0.018	0.033	0.635	0.058	13.31	355.11	21.14
45.50	1.004	6179	0.022	0.041	0.627	0.087	10.19	230.05	20.22

$$R_\sigma = \sum(\sigma(I))/\sum(I) = 0.0276$$

```
                    Residuals after cycle  18

R{ F } =      0.0268                              Rw{ F } =     0.0142
R{F^2} =      0.0221                              Rw{F^2} =     0.0271
 GOFw  =      0.8807          GOF =      0.9940    Nref/Nv =    27.6013
--------------------------------------------------------------------------------
    Mean(Shift/su)  = 0.620871E-06
    Max(Shift/su)   = 0.826861E-04 for variable        1/KS
--------------------------------------------------------------------------------
              Atom-Group(s)  (net charge)
 1 (-0.00020)
--------------------------------------------------------------------------------
   Isotropic Extinction Type_1 Lorentzian Distribution Mosaic Spread_0

         Mosaic Spread (seconds):           Domain Size (centimeters):
                   0.657811E+03                        0.354657E-06
--------------------------------------------------------------------------------

         After cycle 18 convergence criterion was met
         Proceed to last cycle to finalise outputs
--------------------------------------------------------------------------------
--------------------------------------------------------------------------------
--------------------------------------------------------------------------------

 Number of data              =  12807
 Rejected based on OBS       =      0
 Rejected based on SIGOBS    =      0
 Rejected based on SINTHL    =      0
 Total number of rejections  =      0
 Included in the refinement  =  12807

                    Residuals after final cycle

R{ F } =      0.0268                              Rw{ F } =     0.0142
R{F^2} =      0.0221                              Rw{F^2} =     0.0271
 GOFw  =      0.8807          GOF =      0.9940    Nref/Nv =    27.6013
--------------------------------------------------------------------------------
```

Figure S3: MM refinement results for **2**.

Refinement was conducted with all intensity data. The final Multipole refinements on F^2 comprised multipoles up to hexadecapoles for non-H atoms and up to bond–directed dipoles for the H atoms. In the MM, C–H distances were constrained to 1.09 Å.

Chemical constrains for C1, C4;
C2, C3, C5, C6;
C11,C12,C13,C14,C15,C16;
all H-atome.

Contraction parameters κ for non-H atoms were refined freely in a step-wise approach; $\kappa' = 1.0$ for non-H atoms, $\kappa' = 1.2$ for H atoms.

Contraction parameters and multipole population coefficients (see Table S4)

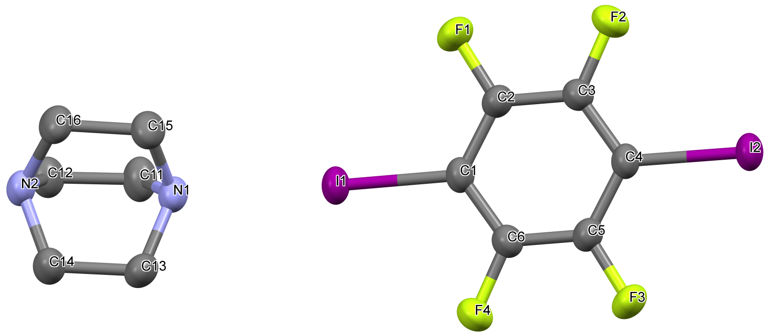

Figure S4: Displacement ellipsoid plot of TFDIB DABCO aggregate in **2** at the 90% probability level; hydrogen atoms omitted.

Table S5: Bond distances (Å) for **2**.

N1···I1	2.7374(11)	N2^i···I2	2.7453(11)
I1—C1	2.1134(10)	I2—C4	2.1119(10)
F1—C2	1.3428(18)	F2—C3	1.3473(19)
F3—C5	1.3390(17)	F4—C6	1.3434(19)
C1—C2	1.3892(13)	C1—C6	1.3884(13)
C2—C3	1.3864(14)	C5—C6	1.3879(13)
C3—C4	1.3905(13)	C4—C5	1.3918(13)
N1—C11	1.4707(15)	N1—C13	1.4764(15)
N1—C15	1.4746(15)	N2—C12	1.4721(15)
N2—C14	1.4734(14)	N2—C16	1.4744(14)
C11—C12	1.5513(14)	C13—C14	1.5507(14)
C15—C16	1.5494(14)		

Table S6: Bond angles (°) for **2**.

I1—C1—C2	121.76(7)
I1—C1—C6	121.22(7)
C2—C1—C6	116.84(8)
F1—C2—C1	120.31(10)
F2—C3—C2	118.09(9)
C11—N1—C15	109.08(9)
C13—N1—C15	108.52(9)
C12—N2—C16	108.57(9)
C16—N2—C14	109.18(9)
N1—C11—C12	109.89(9)
N2—C12—C11	110.11(8)
N2—C14—C13	110.01(8)
N1—C13—C14	109.91(8)

Table S7: Angles (°) of intermolecular interactions for **2**.

N1···I1—C1	173.21(4)
N2^i···I2—C4	173.98(4)
F1···H15A^{ii}—C15^{ii}	131
F2···H16A^{iii}—C16^{iii}	172
F4···H12B^{iv}—C12^{iv}	156
C5—F3···F3^v	96.77(10)

$i = -2+x, -1+y, z; ii = 2-x, 1-y, 2-z; iii = -1+x, -1+y, z; iv = 2-x, 1-y, 1-z; v = -x, -y, 1-z$

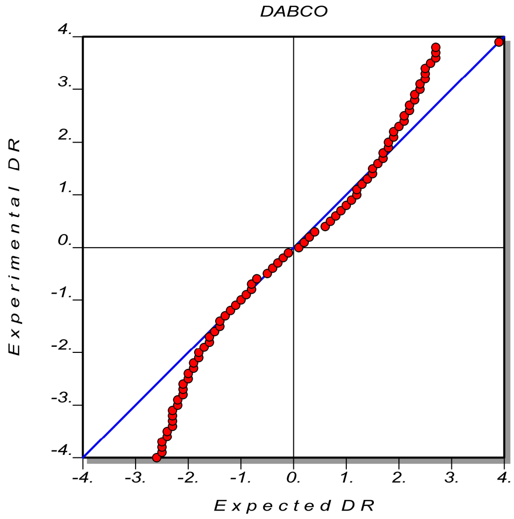

Figure S5: DRplot for **2**.

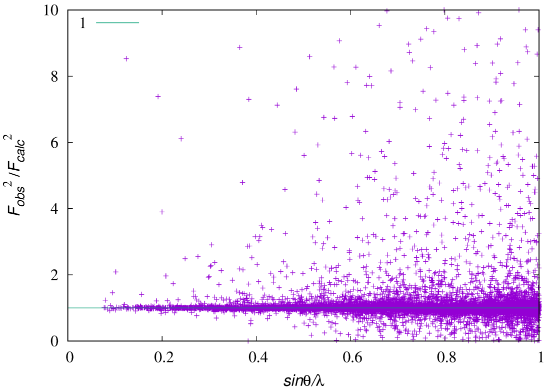

Figure S6: Scatterplot F^2_{obs}/F^2_{calc} vs $sin\theta/\lambda$ for X-ray refinement result (MM) for **2**.

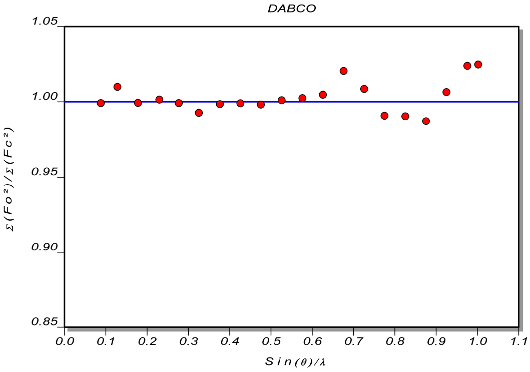

Figure S7: Averaged $K = \sum(F^2_{obs})/\sum(F^2_{calc})$ vs $sin\theta/\lambda$ for X-ray refinement result (MM) for **2**.

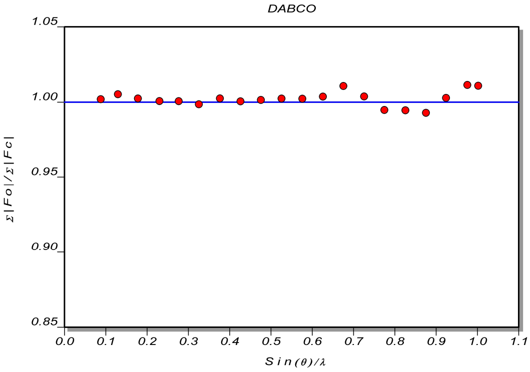

Figure S8: Averaged $K = \sum(|F_{obs}|)/\sum(|F_{calc}|)$ vs $sin\theta/\lambda$ for X-ray refinement result (MM) for **2**.

4.1 Residual electron density distribution

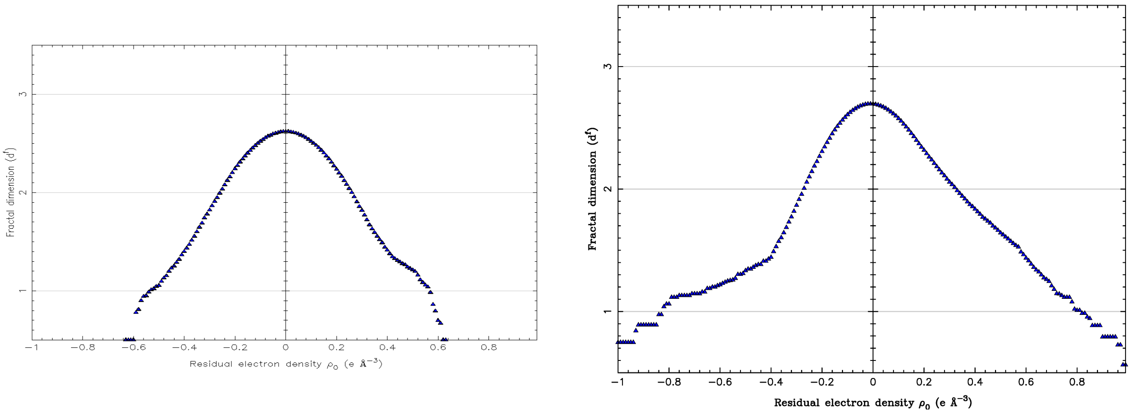

(a) MM

(b) IAM

Figure S9: Fractal dimension plots [1, 2] vs residual electron density.

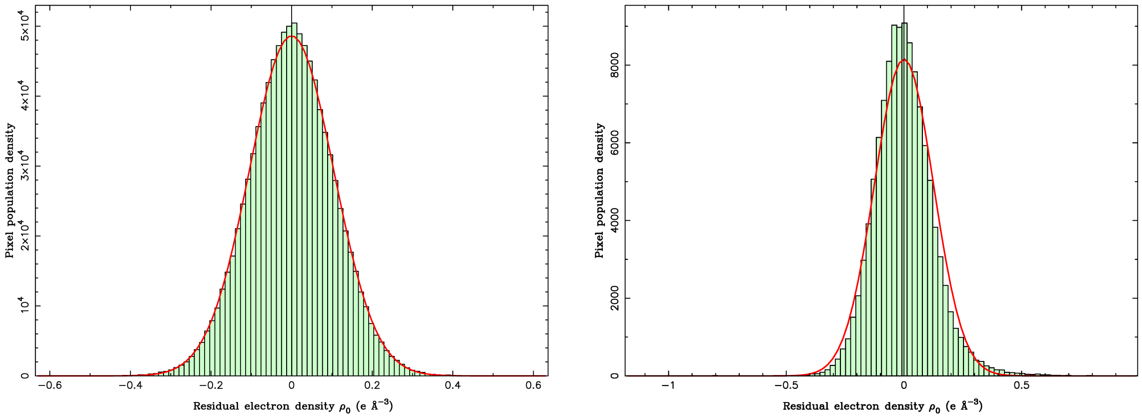

(a) MM

(b) IAM

Figure S10: Probability distribution histogram [1, 2] vs residual electron density.

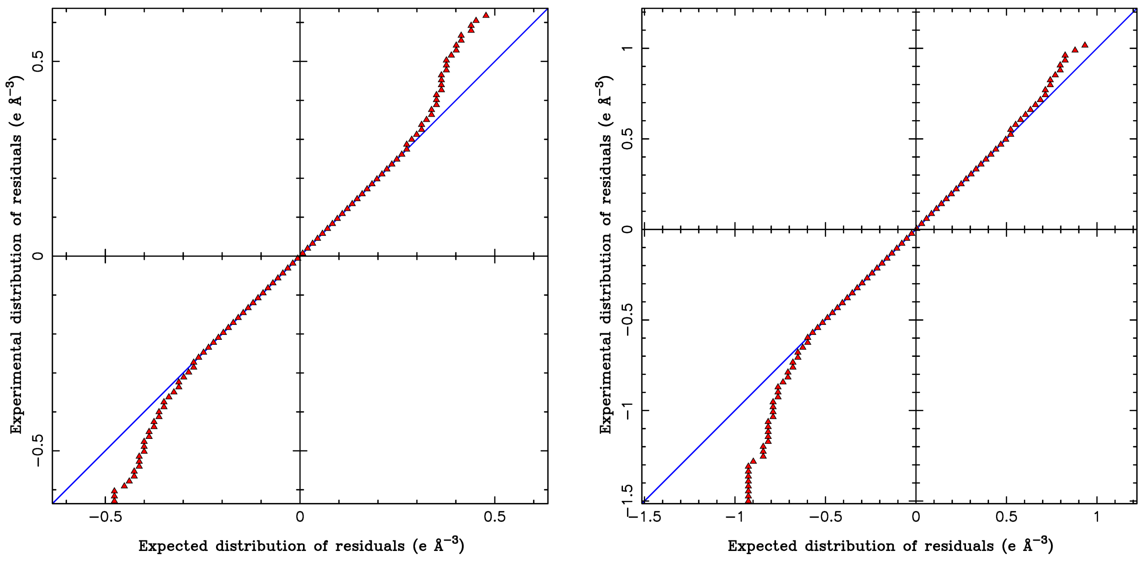

(a) MM

(b) IAM

Figure S11: Normal probability plots [1, 2].

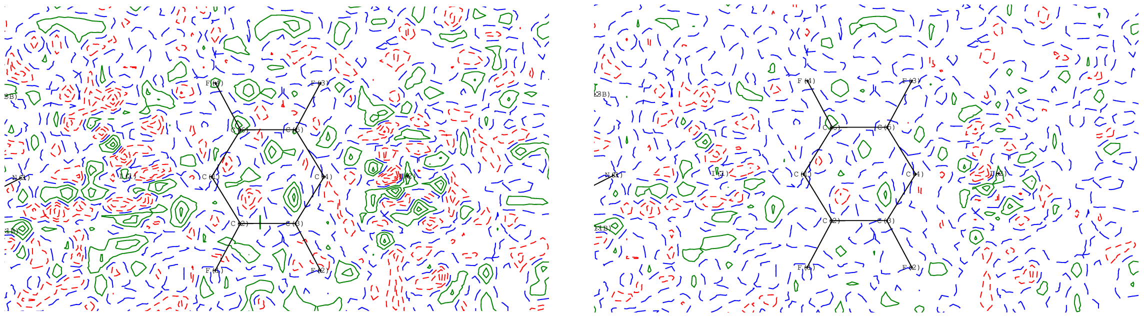

(a) contour 0.1 $e{\cdot}\text{Å}^{-3}$

(b) contour 0.15 $e{\cdot}\text{Å}^{-3}$

Figure S12: Residual electron density after the multipole refinement.
Green: positive
red: negative
blue: zero

5 Topological analysis (AIM)

5.1 Bond critical points

Figure S13: xdprop bcp part results for **2**.

```
      Bond            f            del2f         Rij     d1      d2
                                                 Hessian Eigenvalues    ellip
I(1)    -N(1)         0.229(  0)     2.073(  0)  2.7351 1.5256 1.2095
                                                   -0.60  -0.59   3.26   0.02

      Bond            f            del2f         Rij     d1      d2
                                                 Hessian Eigenvalues    ellip
I(2)    -X1_N(2)      0.226(  0)     2.047(  0)  2.7438 1.5305 1.2133
                                                   -0.59  -0.58   3.21   0.02

      Bond            f            del2f         Rij     d1      d2
                                                 Hessian Eigenvalues    ellip
I(1)    -C(1)         0.701(  0)     3.178(  0)  2.1149 1.1844 0.9305
                                                   -2.28  -2.23   7.69   0.02

      Bond            f            del2f         Rij     d1      d2
                                                 Hessian Eigenvalues    ellip
I(2)    -C(4)         0.703(  0)     3.180(  0)  2.1133 1.1836 0.9298
                                                   -2.29  -2.24   7.70   0.02

Searching internuclear distances between  1.200 and  2.000 Angstroms

      Bond            f            del2f         Rij     d1      d2
                                                 Hessian Eigenvalues    ellip
F(1)    -C(2)         1.728(  0)     1.169(  0)  1.3482 0.8092 0.5389
                                                   -9.52  -9.26  19.95   0.03
F(2)    -C(3)         1.726(  0)     1.192(  0)  1.3487 0.8093 0.5394
                                                   -9.51  -9.25  19.96   0.03
F(3)    -C(5)         1.741(  0)     0.969(  0)  1.3439 0.8090 0.5349
                                                   -9.58  -9.31  19.86   0.03
F(4)    -C(6)         1.724(  0)     1.239(  0)  1.3496 0.8093 0.5403
                                                   -9.50  -9.24  19.98   0.03
N(1)    -C(11)        1.404(  0)     3.110(  0)  1.4721 0.8015 0.6706
                                                   -6.98  -6.94  17.03   0.01
N(1)    -C(13)        1.395(  0)     3.203(  0)  1.4756 0.8027 0.6729
                                                   -6.93  -6.88  17.01   0.01
```

(a) IAM

```
      Bond            f            del2f         Rij     d1      d2
                                                 Hessian Eigenvalues    ellip
I(1)    -N(1)         0.177(  6)     1.984(  3)  2.7388 1.4436 1.2952
                                                   -0.42  -0.35   2.76   0.20

      Bond            f            del2f         Rij     d1      d2
                                                 Hessian Eigenvalues    ellip
I(2)    -X1_N(2)      0.158(  6)     1.947(  3)  2.7439 1.4241 1.3198
                                                   -0.34  -0.27   2.55   0.26

      Bond            f            del2f         Rij     d1      d2
                                                 Hessian Eigenvalues    ellip
I(1)    -C(1)         0.603( 17)     4.846( 13)  2.1145 1.1509 0.9636
                                                   -2.13  -1.66   8.64   0.28

      Bond            f            del2f         Rij     d1      d2
                                                 Hessian Eigenvalues    ellip
I(2)    -C(4)         0.610( 17)     5.112( 14)  2.1146 1.1395 0.9751
                                                   -2.13  -1.68   8.92   0.27

Searching internuclear distances between  1.200 and  2.000 Angstroms

      Bond            f            del2f         Rij     d1      d2
                                                 Hessian Eigenvalues    ellip
F(1)    -C(2)         1.459( 55)     9.384(181)  1.3492 0.8275 0.5216
                                                   -8.34  -5.48  23.20   0.52
F(2)    -C(3)         1.499( 55)     8.929(177)  1.3569 0.8234 0.5336
                                                   -9.29  -6.56  24.77   0.42
F(3)    -C(5)         1.363( 56)    11.810(190)  1.3501 0.8434 0.5067
                                                   -7.74  -5.04  24.59   0.54
F(4)    -C(6)         1.473( 58)     9.163(203)  1.3444 0.8380 0.5064
                                                   -9.09  -5.62  23.87   0.62
N(1)    -C(11)        1.485( 23)     4.342( 22)  1.4730 0.7870 0.6860
                                                   -8.47  -7.85  20.66   0.08
N(1)    -C(13)        1.460( 20)     4.794(  6)  1.4786 0.7955 0.6831
                                                   -8.13  -7.44  20.37   0.09
```

(b) up to l=1

```
        Bond              f            del2f         Rij      d1      d2
                                                     Hessian Eigenvalues    ellip
I(1)     -N(1)          0.173(   9)     2.197(  3)  2.7399 1.4055 1.3345
                                                      -0.39  -0.34   2.93    0.15

        Bond              f            del2f         Rij      d1      d2
                                                     Hessian Eigenvalues    ellip
I(2)     -X1_N(2)       0.159(   9)     2.199(  4)  2.7518 1.3915 1.3603
                                                      -0.37  -0.28   2.84    0.33

        Bond              f            del2f         Rij      d1      d2
                                                     Hessian Eigenvalues    ellip
I(1)     -C(1)          0.658( 21)      4.914( 29)  2.1148 1.1126 1.0022
                                                      -2.52  -2.42   9.85    0.04

        Bond              f            del2f         Rij      d1      d2
                                                     Hessian Eigenvalues    ellip
I(2)     -C(4)          0.672( 21)      5.396( 31)  2.1154 1.0913 1.0241
                                                      -2.74  -2.49  10.63    0.10

Searching internuclear distances between  1.200 and  2.000 Angstroms

        Bond              f            del2f         Rij      d1      d2
                                                     Hessian Eigenvalues    ellip
F(1)     -C(2)          2.053( 59)    -18.505(265)  1.3457 0.7859 0.5598
                                                     -18.77 -16.27  16.54    0.15
F(2)     -C(3)          2.117( 59)    -14.875(256)  1.3488 0.7713 0.5775
                                                     -18.47 -16.62  20.21    0.11
F(3)     -C(5)          1.806( 69)    -12.594(355)  1.3399 0.8537 0.4861
                                                     -14.84 -13.47  15.71    0.10
F(4)     -C(6)          2.063( 62)    -19.731(290)  1.3421 0.7917 0.5505
                                                     -18.76 -16.99  16.02    0.10
N(1)     -C(11)         1.821( 29)    -11.078( 60)  1.4717 0.8155 0.6561
                                                     -13.77 -12.91  15.60    0.07
N(1)     -C(13)         1.811( 25)    -10.841( 33)  1.4773 0.8331 0.6442
                                                     -13.18 -12.86  15.20    0.02
```

(c) up to l=3

```
        Bond              f            del2f         Rij      d1      d2
                                                     Hessian Eigenvalues    ellip
I(1)     -N(1)          0.187( 10)      2.071(  5)  2.7511 1.4560 1.2951
                                                      -0.72  -0.33   3.12    1.17

        Bond              f            del2f         Rij      d1      d2
                                                     Hessian Eigenvalues    ellip

        Bond              f            del2f         Rij      d1      d2
                                                     Hessian Eigenvalues    ellip
I(1)     -C(1)          0.695( 25)      4.715( 49)  2.1147 1.1300 0.9848
                                                      -3.26  -2.11  10.09    0.54

        Bond              f            del2f         Rij      d1      d2
                                                     Hessian Eigenvalues    ellip
I(2)     -C(4)          0.694( 25)      4.610( 39)  2.1200 1.1391 0.9809
                                                      -3.45  -1.97  10.03    0.75

Searching internuclear distances between  1.200 and  2.000 Angstroms

        Bond              f            del2f         Rij      d1      d2
                                                     Hessian Eigenvalues    ellip
F(1)     -C(2)          2.032( 49)    -18.897(209)  1.3438 0.7860 0.5578
                                                     -20.47 -14.99  16.56    0.37
F(2)     -C(3)          2.072( 37)    -22.624( 86)  1.3475 0.8059 0.5416
                                                     -19.68 -18.36  15.42    0.07
F(3)     -C(5)          2.080( 36)    -22.244( 80)  1.3390 0.8045 0.5345
                                                     -19.98 -17.65  15.38    0.13
F(4)     -C(6)          2.129( 35)    -26.829( 77)  1.3449 0.8185 0.5264
                                                     -21.50 -18.88  13.55    0.14
N(1)     -C(11)         1.849( 39)    -14.344(125)  1.4708 0.8217 0.6490
                                                     -14.50 -13.77  13.93    0.05
N(1)     -C(13)         1.875( 40)    -14.035(113)  1.4773 0.8119 0.6654
                                                     -15.46 -13.38  14.81    0.16
```

(d) dup to l=4

Surprise: by searching *bcp* between I2$\cdots$N2^i, no success, because it is not convergent.

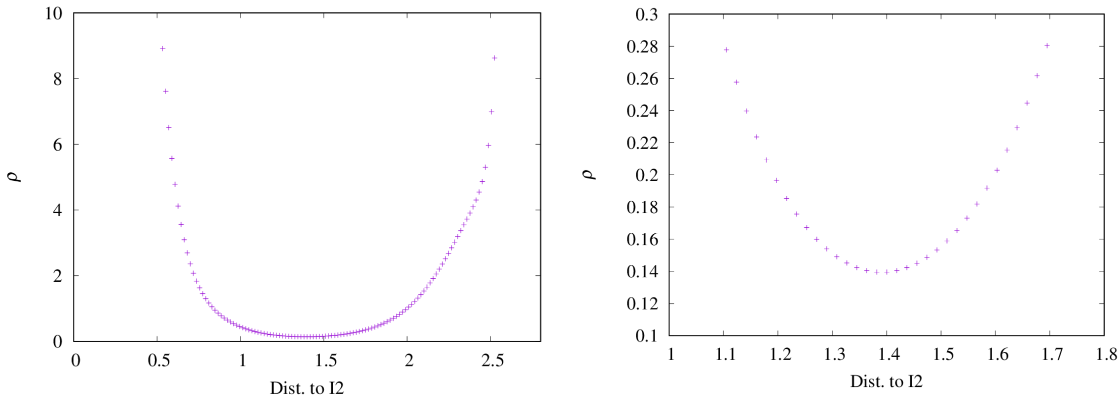

Figure S14: I2$\cdots$N2^i electron density ρ (e/Å^3) distribution for **2**.

```
Searching between atoms I(2)   and X1_N(2)

  Step        x         y         z           |Grad(f)|      Max. step-size

    0     -2.9363 -3.5455  8.2035        1.837E-01
    1     -2.8863 -3.5955  8.2535        1.652E-01          0.05000
    2     -2.8363 -3.6455  8.3035        1.332E-01          0.05000
    3     -2.7863 -3.6955  8.3535        9.313E-02          0.05000
    4     -2.7363 -3.7455  8.4035        5.398E-02          0.05000
    5     -2.6863 -3.7955  8.4535        3.885E-02          0.05000
    6     -2.6363 -3.8455  8.4645        5.120E-02          0.05000
    7     -2.5863 -3.8955  8.4145        5.823E-02          0.05000
    8     -2.5363 -3.9455  8.3645        7.072E-02          0.05000
    9     -2.5863 -3.8955  8.4145        5.823E-02          0.05000
   10     -2.5613 -3.9205  8.3895        6.403E-02          0.02500
   11     -2.5863 -3.8955  8.4145        5.823E-02          0.02500
   12     -2.5738 -3.9080  8.4020        6.099E-02          0.01250
```

found by search on a grid of 0.300 Å

```
16 critical point(s) found, function value =   0.158996
Bond Critical Point   16 connects atoms I(2) and X1_N(2)
Bond path to I(2) =        1.5145 Angstroms
Bond path to X1_N(2) =        1.3316 Angstroms
Total bond path =        2.8461 Angstroms
Distance         =        2.7453 Angstroms
Ratio            =        1.0367
                  property increases...
                  nsteps =         5    pathlength =     0.016841 Angstroms
                  nsteps =        45    pathlength =     4.115783 Angstroms
                  nsteps =        10    pathlength =     1.330600 Angstroms
                  nsteps =         9    pathlength =     1.513574 Angstroms
```

5.2 Topological properties in the bond critical points of the experimental electron density

Table S8: Topological properties in the bond critical points of the experimental electron density.

Bond	dist.(Å)	R_{ij}(Å)	d_1(Å)	$\rho(e\cdot$Å$^{-3})$	$\nabla^2\rho(e\cdot$Å$^{-5})$	$G(a.u.)$	$\frac{G}{\rho}(a.u.)$	$V(a.u.)$	$\frac{\lvert V\rvert}{G}$	$E(a.u.)$
I1···N1	2.7374(11)	2.7616	1.4660	0.19(2)	2.071(5)	0.0216	0.78	–0.0217	1.00	–0.0001
calc.		2.7374	1.4144	0.229	1.716	0.0222	0.65	–0.0267	1.20	–0.0045
I2···N2^{i}	2.7453(11)	2.8461	1.5145	0.16(2)	1.807(5)	0.0181	0.77	–0.0174	0.96	0.0007
calc.		2.7453	1.4158	0.228	1.668	0.0217	0.64	–0.0261	1.20	–0.0044
F1···H15A^{ii}	2.59	2.6176	1.4785	0.038(2)	0.553(2)	0.0043	0.77	–0.0029	0.67	0.0014
F2···H16A^{iii}	2.47	2.4722	1.4911	0.038(2)	0.708(2)	0.0054	0.96	–0.0035	0.65	0.0019
F4···H12B^{iv}	2.41	2.4193	1.4421	0.046(2)	0.836(2)	0.0065	0.95	–0.0043	0.66	0.0022
F3···F3^{v}	2.893(2)	2.8958	1.4658	0.045(2)	0.765(4)	0.0060	0.89	–0.0040	0.67	0.0020
I1—C1		2.1147	1.1300	0.69(3)	4.72(5)					
I2—C4		2.1200	1.1391	0.69(3)	4.61(4)					
F1—C2		1.3438	0.7860	2.03(5)	–18.9(3)					
F2—C3		1.3475	0.8059	2.07(4)	–22.62(9)					
F3—C5		1.3390	0.8045	2.08(4)	–22.24(8)					
F4—C6		1.3449	0.8185	2.13(4)	–26.83(8)					
C1—C2		1.3891	0.6690	2.18(4)	–21.8(2)					
C2—C3		1.3883	0.6943	2.22(2)	–21.70(8)					
N1—C11		1.4708	0.8217	1.85(4)	–14.3(2)					
N2—C12		1.4730	0.8185	1.95(4)	–18.0(2)					
C11—C12		1.5517	0.7863	1.70(3)	–15.88(6)					

$i = -2+x, -1+y, z; ii = 2-x, 1-y, 2-z; iii = -1+x, -1+y, z; iv = 2-x, 1-y, 1-z; v = -x, -y, 1-z$

Topological properties of (3, -1) critical points for the most relevant intermolecular interactions; d_1 (d_2) is the Bondpath lange from the first (second) atom to the (3, -1) critical point, $R_{ij} = d_1 + d_2$, ρ is the electron density, $\nabla^2\rho$ is the Laplacian of the electron density. $G(a.u.)$ is the kinetic energy density, $G/\rho(a.u.)$ the ratio between kinetic energy density and electron density, $V(a.u.)$ the potential energy density and $E(a.u.)$ the total energy density in the bond critical point [4, 5].

6 Theoretical calculations

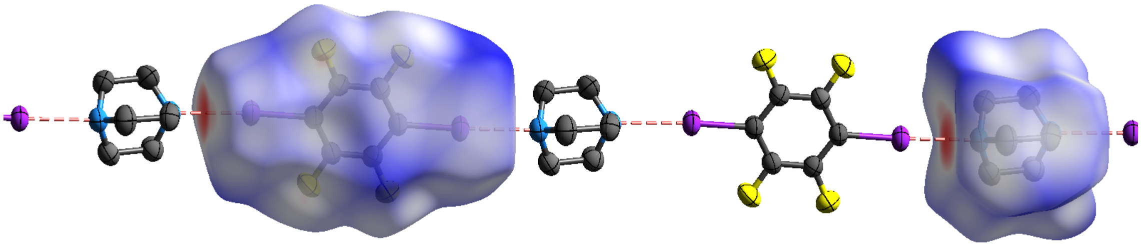

Figure S15: Short N$\cdots$I contact in the aggregate in **2**; Hirshfeld surface is shown for the TFDIB and DABCO moiety. The surface colours code the electrostatic potential (red negative, blue positive) [6].

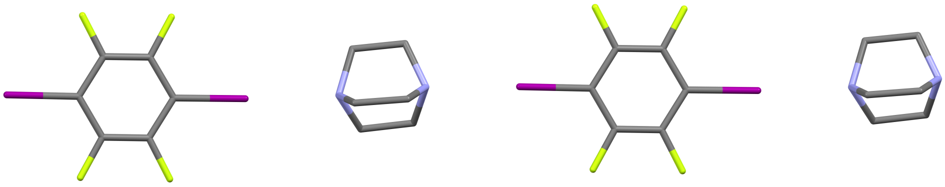

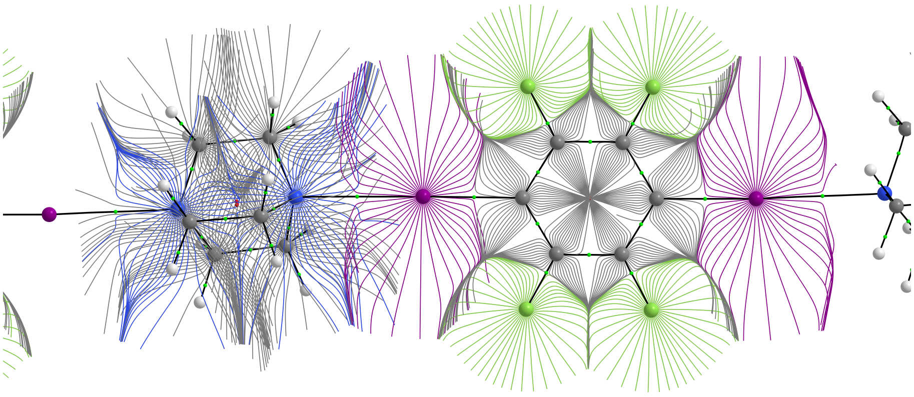

Figure S16: $|\nabla\rho|$ from calculation; bond paths are shown as black lines, bond critical points as black solid circles, and ring critical points as green solid circles.[7]
Fig. S16 shows a trajectory plot an isolated four molecular aggregate at the B3LYP level of theory.

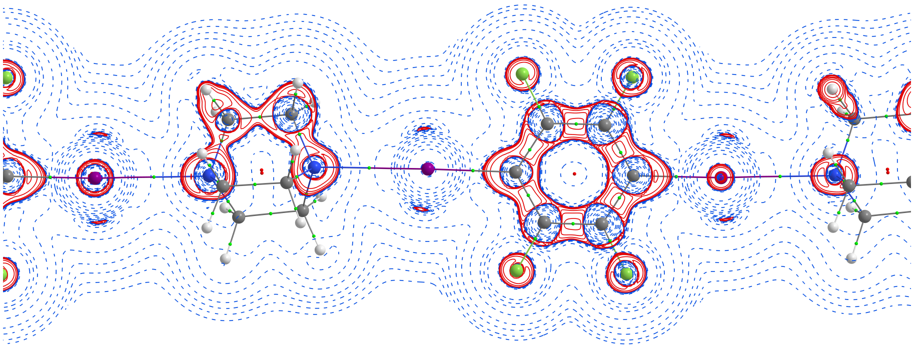

Figure S17: Laplacian of the electron density obtained by calculation ($\nabla^2\rho$); contours are at $\pm 2^n \cdot 10^{-3}$ a.u. Positive values are in blue and negative values in red **2**.

References

[1] K. Meindl and J. Henn. Foundations of Residual-Density Analysis. *Acta Crystallogr.* **2008**, *A64*, 404–418.

[2] A. Volkov, P. Macchi, L. J. Farrugia, C. Gatti, P. Mallinson, T. Richter, and T. Koritsanszky. *XD2016 - A Computer Program Package for Multipole Refinement, Topological Analysis of Charge Densities and Evaluation of Intermolecular Energies from Experimental and Theoretical Structure Factors.* **2016**.

[3] C. B. Hübschle and B. Dittrich. *MoleCoolQt* - a molecule viewer for charge-density research. *J. Appl. Crystallogr.*, 44:238–240, 2011.

[4] Y. A. Abramov. On the possibility of kinetic energy density evaluation from the experimental electron-density distribution. *Acta Crystallogr.*, A53:264–272, 1997.

[5] E. Espinosa, E. Molins, and C. Lecomte. Hydrogen bond strengths revealed by topological analyses of experimentally observed electron densities. *Chem. Phys. Lett.*, 285:170–173, 1998.

[6] M. J. Turner, J. J. McKinnon, S. K. Wolff, D. J. Grimwood, P. R. Spackman, D. Jayatilaka, and M. A. Spackman. *CrystalExplorer17* (Version 17.5). University of Western Australia, http://hirshfeldsurface.net, 2017.

[7] T. A. Keith. *AIMAll (Version 17.01.25).* TK Gristmill Software, Overland Park KS, USA, 2017.

Table S4: Contraction parameters and Population coefficients

atom	P_v	κ	P_{00}	P_{11}	P_{1-1}	P_{10}	Net charge
I(1)	7.12(8)	1.049(4)	0	0.13(3)	0.00(3)	–0.07(2)	–0.12(8)
I(2)	7.15(8)	1.049(4)	0	0.01(3)	–0.01(3)	–0.02(3)	–0.15(8)
F(1)	7.19(3)	0.984(3)	0	–0.01(4)	–0.02(3)	–0.04(3)	–0.19(3)
F(2)	7.19(3)	0.984(3)	0	0.07(3)	0.02(3)	0.01(3)	–0.19(3)
F(3)	7.22(3)	0.984(3)	0	–0.02(3)	0.03(3)	–0.03(2)	–0.22(3)
F(4)	7.21(3)	0.984(3)	0	–0.10(3)	0.00(3)	–0.06(3)	–0.21(3)
N(1)	5.07(6)	0.993(5)	0	0.10(2)	–0.03(2)	–0.04(2)	–0.07(6)
N(2)	5.00(6)	0.993(5)	0	0.11(2)	–0.04(2)	–0.03(2)	0.00(6)
C(1)	4.23(10)	0.993(5)	0	–0.01(2)	0.03(2)	0.03(2)	–0.23(10)
C(2)	3.93(6)	1.002(6)	0	–0.03(2)	0.02(2)	–0.12(2)	+0.07(6)
C(11)	4.18(5)	0.987(5)	0	–0.01(2)	0.06(2)	0.04(2)	–0.18(5)
H(11A)	0.80(2)	1.2	0	0	0	0.11(2)	+0.20(2)

atom	P_{20}	P_{21}	P_{2-1}	P_{22}	P_{2-2}
I(1)	–0.45(3)	–0.06(3)	0.02(3)	–0.15(3)	0.07(3)
I(2)	–0.52(3)	0.09(3)	0.04(3)	–0.16(3)	–0.10(3)
F(1)	–0.12(3)	–0.05(3)	–0.14(3)	0.02(4)	0.05(4)
F(2)	–0.13(3)	–0.05(3)	–0.05(3)	0.01(3)	0.01(3)
F(3)	–0.07(3)	0.04(3)	–0.02(3)	–0.08(3)	–0.02(3)
F(4)	–0.05(3)	0.00(3)	–0.08(3)	0.00(3)	–0.06(3)
N(1)	0.00(2)	–0.03(2)	0.04(2)	0.00(2)	–0.06(2)
N(2)	–0.02(2)	0.00(2)	–0.01(2)	0.06(2)	–0.06(2)
C(1)	0.07(2)	0.05(2)	0.04(2)	–0.11(2)	–0.04(2)
C(2)	0.03(2)	–0.01(2)	0.03(2)	–0.17(2)	0.04(2)
C(11)	0.04(2)	0.02(2)	0.00(2)	–0.07(2)	–0.01(2)

atom	P_{30}	P_{31}	P_{3-1}	P_{32}	P_{3-2}	P_{33}	P_{3-3}
I(1)	0.05(3)	0.02(2)	0.02(2)	0.01(3)	0.00(2)	0.03(2)	–0.01(2)
I(2)	0.09(3)	–0.03(2)	0.05(2)	0.00(3)	0.04(2)	–0.04(2)	–0.03(2)
F(1)	0.00(2)	0.00(2)	–0.01(2)	0.06(2)	0.03(2)	–0.01(2)	–0.01(2)
F(2)	0.00(2)	–0.05(2)	0.02(2)	0.00(2)	0.00(2)	–0.02(2)	–0.01(2)
F(3)	0.01(2)	–0.03(2)	–0.02(2)	0.01(2)	0.01(2)	–0.02(2)	0.05(2)
F(4)	–0.03(2)	0.02(2)	–0.05(2)	0.03(2)	0.04(2)	–0.04(2)	0.02(2)
N(1)	0.18(2)	0.04(2)	0.00(2)	0.04(2)	–0.07(2)	0.04(2)	–0.12(2)
N(2)	0.19(2)	0.01(2)	0.01(2)	0.00(2)	–0.04(2)	–0.02(2)	–0.10(2)
C(1)	0.22(3)	0.03(2)	–0.01(2)	0.16(2)	0.00(2)	0.03(2)	0.02(2)
C(2)	0.33(2)	–0.02(2)	0.01(2)	0.12(2)	–0.04(3)	0.00(2)	0.01(2)
C(11)	0.29(2)	–0.03(2)	0.04(2)	–0.04(2)	–0.01(2)	0.01(2)	–0.23(2)

atom	P_{40}	P_{41}	P_{4-1}	P_{42}	P_{4-2}	P_{43}	P_{4-3}	P_{44}	P_{4-4}
I(1)	0.11(3)	–0.10(3)	0.01(3)	0.20(3)	0.19(3)	0.00(3)	–0.09(3)	0.11(3)	0.18(3)
I(2)	0.06(3)	0.18(3)	0.04(3)	0.14(3)	–0.19(3)	0.02(3)	–0.11(3)	–0.03(3)	–0.18(3)
F(1)	–0.03(3)	0.04(3)	–0.03(3)	0.10(3)	–0.04(3)	0.04(3)	0.02(3)	–0.09(3)	0.04(3)
F(2)	0.00(3)	–0.06(3)	–0.09(3)	–0.02(3)	0.05(3)	–0.01(3)	–0.04(3)	0.01(3)	–0.01(3)
F(3)	–0.03(3)	–0.01(3)	–0.10(2)	0.00(3)	0.12(3)	0.06(3)	–0.02(3)	0.01(3)	–0.07(3)
F(4)	0.06(3)	0.04(3)	0.03(3)	0.01(3)	0.00(3)	–0.03(3)	0.03(3)	–0.02(3)	–0.06(3)
N(1)	–0.01(3)	–0.04(3)	0.01(3)	–0.01(3)	–0.05(3)	0.00(3)	–0.04(3)	–0.03(2)	–0.02(3)
N(2)	0.04(3)	–0.02(3)	0.01(3)	–0.01(3)	–0.01(3)	0.01(3)	0.03(3)	0.01(2)	0.11(3)
C(1)	0.08(3)	0.07(2)	0.09(3)	0.00(3)	0.01(4)	0.01(2)	0.09(2)	0.00(2)	0.06(2)
C(2)	0.10(2)	0.00(2)	0.06(2)	0.04(2)	0.06(3)	–0.03(2)	–0.02(2)	0.03(2)	–0.03(2)
C(11)	0.03(2)	–0.05(2)	–0.04(2)	–0.02(2)	0.02(2)	–0.04(2)	0.12(2)	–0.01(2)	0.00(2)
